# Evolution of the genus *Eucricetodon* (Rodentia, Mammalia) from the Valley of Lakes (Mongolia): a taxonomical description and update on the stratigraphical distribution

**DOI:** 10.1007/s12549-016-0256-x

**Published:** 2016-11-22

**Authors:** Paloma López-Guerrero, Olivier Maridet, Gudrun Daxner-Höck

**Affiliations:** 1 Geologisch-Paläontologische Abteilung, Naturhistorisches Museum Wien, Burgring 7, 1010 Vienna, Austria; 2 Jurassica Museum, Collection Management Center, Route de Fontenais 21, 2900 Porrentruy, Switzerland; 3 Department of Geosciences, Earth Sciences, University of Fribourg, Chemin du Musée 6/Pérolles, 1700 Fribourg, Switzerland

**Keywords:** *Eucricetodon*, Mongolia, Oligocene, Ulantatal, China, Systematics

## Abstract

The Oligocene fossil deposits from Valley of Lakes in Central Mongolia have provided a wealth of rodent fossils. Among these, cricetids are a very important part. To date, only the Miocene genera have been described in detail. Here, we focus on the Oligocene genus *Eucricetodon* from this region. Eucricetodontinae are the most abundant fossils in the Oligocene Valley of Lakes faunas. The present study consists of the description of five species of cricetid rodents from 43 localities ranging in age from the early Oligocene to the early-late Oligocene. In addition to *Eucricetodon asiaticus* described in Mongolia in 1923, we have found *Eucricetodon bagus* and *Eucricetodon jilantaiensis* that were described from Nei Mongol and *Eucricetodon occidentalis* discovered in Kazakhstan. This taxonomical study provides new information regarding the evolution of the Cricetidae in Central and Eastern Asia during the Oligocene and, more particularly, regarding their phylogenetic relationships and the evolutionary trends.

## Introduction

The species studied here belong to the genus *Eucricetodon* Thaler, 1966. It was firstly described as a subgenus within *Cricetodon*, and Thaler did not include any original diagnosis on the description. Later, it was erected as genus by Mein and Freudenthal (1971) but Vianey-Liaud (1972) proposed the first diagnosis. The most recent definition is found in Dienemann (1987). More than 25 species have been described under *Eucricetodon* (Wang 1987; Ünay 1989; Maridet et al. 2009; Gomes Rodrigues et al. 2012a; Li et al. 2016). This is one of the eight genera of cricetids known from the Oligocene of Europe (Mein and Freudenthal 1971) and an important member of the Asian assemblages. Diversity of *Eucricetodon* in Asia is poorly constrained compared to that recorded in Europe. It is common in Kazakhstan (Shevyreva 1967; Lopatin 1996) and China (Wang 1987), but before the revision of the Ulantatal material by Gomes Rodrigues et al. (2012a), only a few species of *Eucricetodon* were known in the Asian Oligocene (Lindsay 1978; Wang 1987, 2007; Wang and Qiu 2000; Lopatin 1996). The knowledge of *Eucricetodon* has been recently improved with new discoveries and revisions in Kazakhstan (Bendukidze et al. 2009) as well as in Inner Mongolia (Gomes Rodrigues et al. 2012a; Li et al. 2016) and the Junggar basin (Maridet et al. 2009) in China. The first occurrence of the genus in Mongolia was remarked by Lindsay (1978). He revised the material of *Eucricetodon asiaticus* (Matthew and Granger 1923) from the middle Oligocene of Mongolia which was previously ascribed to the American genus *Eumys* by Matthew and Granger (1923). Nevertheless, the works carried out under the recent Austrian-Mongolian joint projects (1995–2015) in the Valley of Lakes have provided a wealth of fossils (Daxner-Höck et al. 1997, 2015; Höck et al. 1999; Daxner-Höck 2000, 2001; Daxner-Höck and Wu 2003; Schmidt-Kittler et al. 2007; Maridet et al. 2014). The present study is focused on the systematical revision of the *Eucricetodon* from the Taatsiin Gol and Taatsiin Tsagaan Nuur areas in Mongolia (for geological details, see Daxner-Höck et al. 2017, this issue). The presence of *Eucricetodon* in the material recovered in these areas was previously reported (Daxner-Höck et al. 2010; Maridet et al. 2014), but detailed descriptions were lacking until the present paper. It is worth noticed that the taxonomical interpretation has change greatly in comparison with the previous works published. Here, we describe all the species found in the early and early-late Oligocene providing an excellent framework for the study of the morphological changes undergone by this genus during the Oligocene.

## Materials and Methods

Institutional abbreviations

**NHMW:** Naturhistorisches Museum Wien, Vienna, Austria

Locality abbreviations

TAT: Tatal Gol; TGR: Taatsin Gol Rigth; TGL: Taatsin Gol Left; SHG: Hsanda Gol; DEL: Del; IKH: Ikh Argalatyn Nuruu; UNCH: Unkheltseg; ABO: Abzag Ovo; TAR: Unzing Churum; TGW: Torglorhoi

Material

**Fig. 1:**
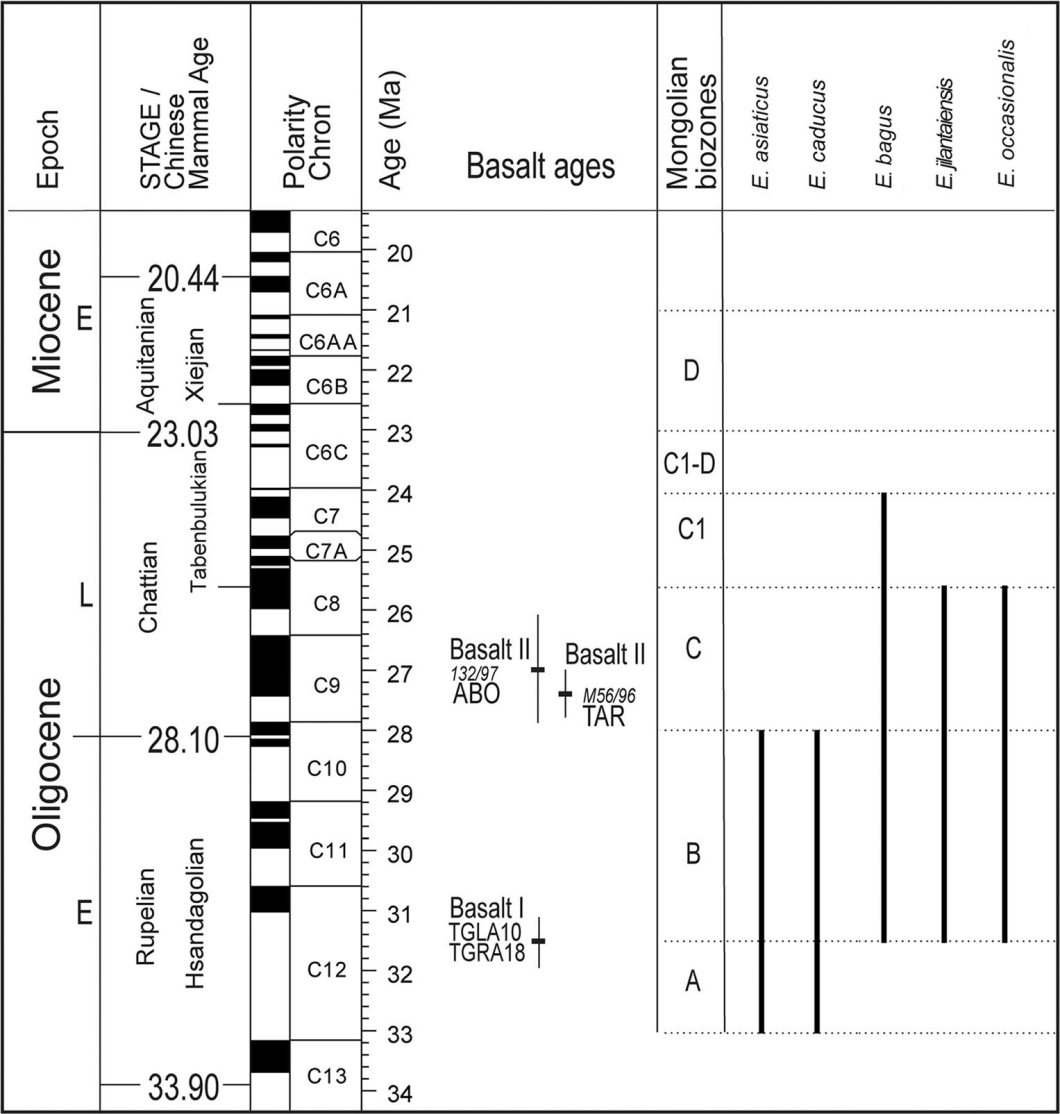
Stratigraphical distributions of the species of *Eucricetodon* found in Mongolia. The stratigraphic chart (modified after Daxner-Höck et al. 2014, 2015) includes the geologic time scale (Vandenberghe et al. 2012), basalt ages and Mongolian biozones A to C1 (Höck et al. 1999), Mongolian mammal assemblages (Daxner-Höck et al. 2014), and magnetostratigraphical data (Sun and Windley 2015)

**Fig. 2:**
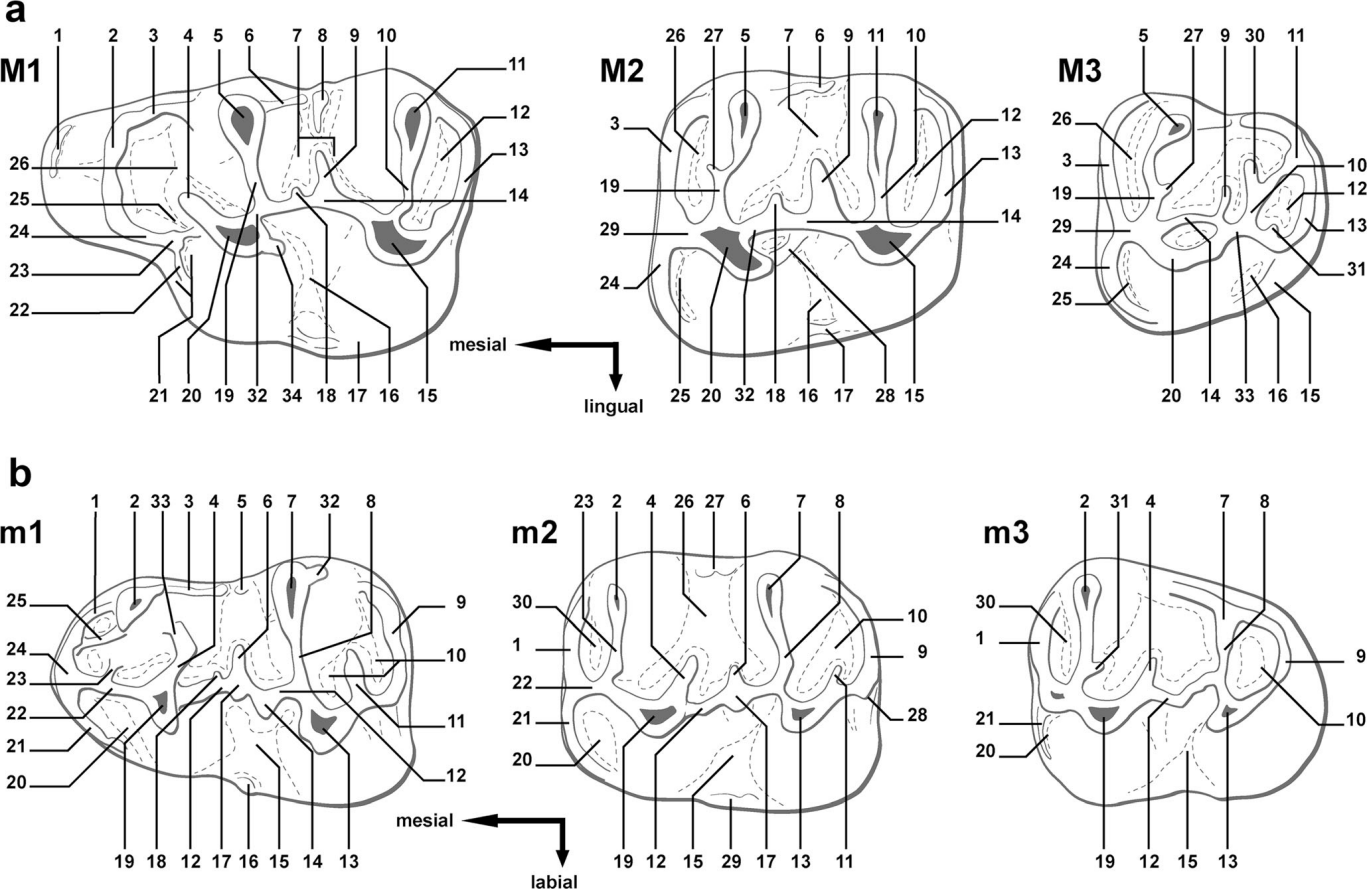
Terminology used in this paper to described molars, modified from Maridet et al. (2009). **a** Upper molars, M1, M2, and M3: *1* anterior crest; *2* anterocone; *3* labial anteroloph; *4* anterior arm of the protocone; *5* paracone; *6* paracone spur; *7* mesosinus; *8* mesostyle; *9* mesoloph; *10* metalophule; *11* metacone; *12* posterosinus; *13* posteroloph; *14* entoloph; *15* hypocone; *16* sinus; *17* lingual cingulum; *18* second mesoloph; *19* protolophule I; *20* protocone; 21 protocone platform; *22* protostyle spur; *23* protostyle; *24* lingual anteroloph; *25* protosinus; *26* anterosinus; *27* protolophule spur; *28* entomesoloph; *29* anterolophule; *30* neomesoloph; *31* neometalophule; *32* posterior arm of the protocone; *33* neoentoloph; *34* protocone spur. **b** Lower molars, m1, m2, and m3: *1* lingual anterolophid; *2* metaconid; *3* metaconid ridge; *4* posterior arm of the protoconid; *5* mesostylid; *6* mesolophid; *7* entoconid; *8* hypolophulid; *9* posterolophid; *10* posterosinusid; *11* hypoconid hind arm; *12* ectolophid; *13* hypoconid; *14* ectomesolophid; *15* sinusoid; *16* ectostylid; *17* mesoconid; *18* second mesolophid; *19* protoconid; *20* protosinusid; *21* labial anterolophid; *22* anterolophulid; *23* metalophulid I; *24* anteroconid; *25* metaconid spur; *26* mesosinusid; *27* lingual cingulum; *28* labial posterolophid; *29* labial cingulum; *30* anterosinusid; *31* metalophulid spur; *32* entoconid spur; *33* metalophulid II

The studied material includes 542 upper and lower molars, from 43 localities of Oligocene age, belonging to five species of the genus *Eucricetodon* Thaler, 1966. Fossil sites are situated in the Taatsiin Gol and TaatsiinTsagaan Nuur areas (Mongolia). The fossils are stored in the collections of the Geological-Paleontological Department at the Museum of Natural History of Vienna (Austria). Table 1 shows the different localities studied, the number of molars examined, and the chronological information (for more details, see Daxner-Höck et al. 2017; Harzhauser et al. 2017, this issue). The sites belong to the local biozones A and B from the early Oligocene and C and C1 from the late Oligocene (Daxner-Höck et al. 2010). These biozones are correlated to the Rupelian (A–B) and Chattian (C–C1) stages (Fig. 1). We have compared the Mongolian material with the collection of Ulantatal section (China) stored at the Institute of Vertebrate Paleontology and Paleoantropology in Beijing (China) and with the casts of the material from Altynshokysu (bone bed 2) from Kazakhstan stored at the NHMW. The terminology used to describe the teeth is taken from Maridet et al. (2009), and it is illustrated in Fig. 2. The anatomical abbreviations for upper molars are M1, M2, and M3 and for lower molars, m1, m2, and m3. The observations and measurements were carried out using a Zeiss Discovery V20 binocular microscope. Maximum length and width measurements for each specimen, given in millimeter, were taken using Carl Zeiss Axiocam MRc5 software by means of a digital camera attached to a microscope. All the measurements are given in Table 2. The photographs were taken with a Philips XL 30 scanning electron microscope at the Core Facility of Cell Imaging and Ultrastructure Research (CIUS) EM LAB, Faculty of Life Sciences, University of Vienna (Austria). We have followed the classifications of Mein and Freudenthal (1971), which include the genus into the subfamily Eucricetodontinae, and Wilson and Reeder (2005) who reviewed the status of the family Cricetidae.

**Table 1:** Studied material of *Eucricetodon* from the Taatsiin Gol and TaatsiinTsagaan Nuur areas

Biozone	Species	Locality	M1	M2	M3	m1	m2	m3	Total
B	*E. asiaticus*	UNCH-A/3		3					**3**
B	*E. asiaticus*	SHG-AB/17-18	3	2	2	2	4	6	**19**
B	*E. asiaticus*	SHG-A/20		2	2	2	2	1	**9**
B	*E. asiaticus*	SHG-A/15+20					1		**1**
B	*E. asiaticus*	TGR-AB/22	2	3	2	1	2	1	**11**
B	*E. asiaticus*	TGR-AB/21	2	1	1	3	2	5	**14**
B	*E. asiaticus*	TGR-B/1	1	5	2	7	3	2	**20**
B	*E. asiaticus*	IKH-A/3-4	4			3	2	1	**10**
B	*E. asiaticus*	IKH-A/2		3		3	3	1	**10**
B	*E. asiaticus*	IKH-A/1					1		**1**
B	*E. asiaticus*	TAT-E/3				2	1		**3**
B	*E. asiaticus*	TGL-A/11c			1	1	1	1	**4**
B	*E. asiaticus*	SHG-A/9				1			**1**
B	*E. asiaticus*	TAT-C/7	1	2	1	2	2		**8**
B	*E. asiaticus*	TAT-C/6		1			1	1	**3**
A	*E. asiaticus*	SHG-C/1	1	2					**3**
A	*E. asiaticus*	TAT-C/3	1	1					**2**
A	*E. asiaticus*	TAT-C/2	1			1	2	2	**6**
A	*E. asiaticus*	TGR-A/13					1		**1**
A	*E. asiaticus*	TAT-D/1		1		1	2	1	**5**
		**Total**	**16**	**26**	**11**	**29**	**30**	**22**	**134**
B	*E. caducus*	UNCH-A/3					3	1	**4**
B	*E. caducus*	SHG-A/20	1						**1**
B	*E. caducus*	SHG-A/15+20	2				1		**3**
B	*E. caducus*	SHG-A/15					1		**1**
B	*E. caducus*	TGR-AB/22					1	1	**2**
B	*E. caducus*	TGR-AB/21					1		**1**
B	*E. caducus*	TGR-ZO/2	1	2					**3**
B	*E. caducus*	TGR-ZO/1					1		**1**
B	*E. caducus*	TGR-B/1	1		1		1		**3**
B	*E. caducus*	IKH-A/2	1				1		**2**
B	*E. caducus*	IKH-A/1		2	1	1			**4**
B	*E. caducus*	TAT-E/3				2	1	1	**4**
B	*E. caducus*	TGL-A/11b		1	1				**2**
B	*E. caducus*	SHG-A/9				1	3		**4**
B	*E. caducus*	TAT-C/7				2	1		**3**
B	*E. caducus*	TAT-C/6			1		1		**2**
B	*E. caducus*	DEL-B/7	1						**1**
A	*E. caducus*	SHG-C/1	2	1		1	2		**6**
A	*E. caducus*	TAT-C/3	1					1	**2**
A	*E. caducus*	TAT-C/2				1			**1**
A	*E. caducus*	TAT-C/1			2				**2**
A	*E. caducus*	TGL-A/2	1	3		2	3		**9**
A	*E. caducus*	TGR-A/14	1	2		1	1	1	**6**
A	*E. caducus*	TGR-A/13	2	1					**3**
A	*E. caducus*	TAT-D/1	11	8	5	12	12	5	**53**
		**Total**	**25**	**20**	**11**	**23**	**34**	**10**	**123**
C1	*E*. cf. *bagus*	TAT-surf				1	1	1	**3**
C1	*E. bagus*	TAT-E/27	1						**1**
C	*E. bagus*	IKH-B/5					1		**1**
C	*E. bagus*	DEL-B/12	1			2	1		**4**
C	*E. bagus*	TGW-A/2b	7	11	5	7	4	3	**37**
C	*E. bagus*	TGW-A/2a	19	9	5	8	3	4	**48**
C	*E. bagus*	TAR-A/2	4	3	1	4	2		**14**
C	*E*. cf. *bagus*	ABO-083					1		**1**
C	*E. bagus*	ABO-A/3	2	2	1	1	1		**7**
C	*E. bagus*	TGR-C/2	6	6	1	10	7	3	**33**
C	*E. bagus*	TGR-C/1	13	13	2	13	15	2	**58**
B	*E. bagus*	UNCH-A/3		1					**1**
B	*E*. cf. *bagus*	IKH-A/3-4		1				1	**2**
B	*E. bagus*	IKH-A/1	1						**1**
B	*E. bagus*	TAT-E/3		1					**1**
		**Total**	**54**	**47**	**15**	**46**	**36**	**14**	**212**
C	*E. jilantaiensis*	TGW-A/2b	7	6		4	6	3	**26**
C	*E. jilantaiensis*	TGW-A/2a	1	4		12	9		**26**
C	*E. jilantaiensis*	TGW-A/1	1						**1**
C	*E. jilantaiensis*	TAR-A/2		1			1		**2**
C	*E. jilantaiensis*	TGR-C/1		2	1	1	3		**7**
B	*E. jilantaiensis*	TGR-AB/21		1					**1**
B	*E. jilantaiensis*	TGR-ZO/2					1		**1**
		**Total**	**9**	**14**	**1**	**17**	**20**	**3**	**64**
B	*E. occasionalis*	TGW-AB/22	2			1		1	**4**
B	*E*. cf. *occasionalis*	IKH-A/2	1	3			1		**5**

Local biozones after Daxner-Höck et al. 2014 (A and B early Oligocene; C and C1 late Oligocene). For the locality abbreviations, see Daxner-Höck et al. 2017 (this issue)

**Table 2:** Lengths and widths of the upper and lower molars taken of *Eucricetodon* species from Valley of Lakes (Mongolia)

		Length				Width				
	***E. asiaticus***	***N***	**Min**	**Mean**	**Max**	***N***	**Min**	**Mean**	**Max**	***L***/***W***
M1	IKH-A/3-4	1	–	2.27	–	1	–	1.68	–	1.350
	TGR-AB/22	2	2.46	2.52	2.59	2	1.72	1.73	1.74	1.458
	TGR-AB/21	2	2.33	2.40	2.47	2	1.65	1.66	1.67	1.447
	TGR-B/1	1	–	2.68	–	1	–	1.74	–	1.540
	TAT-C/7	1	–	2.26	–	1	–	1.51	–	1.497
	TAT-C/3	1	–	2.36	–	1	–	1.68	–	1.404
	TAT-C/2	1	–	2.34	–	1	–	1.69	–	1.389
	SHG-AB/17-18	3	2.27	2.39	2.55	3	1.52	1.57	1.62	1.526
	SHG-C/1	0	–	–	–	1	–	1.76	–	–
M2	UNCH-A/3	1	–	1.83	–	1	–	1.66	–	1.106
	SHG-AB/17-18	2	1.75	1.77	1.79	2	1.58	1.58	1.58	0.112
	SHG-A/20	1	–	1.75	–	1	–	1.58	–	1.104
	TGR-AB/22	2	1.76	1.77	–	2	1.58	1.67	1.77	1.057
	TGR-AB/21	1	–	1.78	–	1	–	1.48	–	1.204
	TGR-B/1	5	1.70	1.80	1.87	4	1.60	1.67	1.72	1.076
	IKH-A/2	3	1.76	1.84	1.89	3	1.67	1.74	1.78	1.056
	TAT-C/7	2	1.81	1.84	1.87	2	1.60	1.68	1.77	1.092
	TAT-C/6	0	–	–	–	1	–	1.72	–	–
	SHG-C/1	2	1.65	1.70	1.75	2	1.55	1.58	1.61	1.077
	TAT-C/3	1	–	1.80	–	1	–	1.68	–	1.075
	TAT-D/1	1	–	1.81	–	1	–	1.66	–	1.089
M3	SHG-AB/17-18	2	1.41	1.45	1.48	2	1.41	1.45	1.49	0.997
	SHG-A/20	2	1.41	1.45	1.48	2	1.39	1.39	1.39	1.043
	TGR-AB/22	1	–	1.43	–	1	–	1.48	–	0.964
	TGR-AB/21	1	–	1.35	–	1	–	1.42	–	0.950
	TGR-B/1	2	1.46	1.57	1.67	2	1.39	1.47	1.55	1.063
	TGR-A/11c	1	–	1.55	–	1	–	1.48	–	1.046
m1	SHG-AB/17-18	2	2.05	2.14	2.24	2	1.40	1.48	1.57	1.444
	SHG-A/20	2	1.88	1.91	1.93	2	1.27	1.28	1.29	1.493
	TGR-AB/22	0	–	–	–	1	–	1.29	–	–
	TGR-AB/21	3	1.86	1.90	1.97	3	1.28	1.37	1.44	1.388
	TGR-B/1	6	2.06	2.09	2.15	7	1.19	1.43	1.59	1.461
	IKH-A/3-4	2	1.87	1.93	2.00	3	1.29	1.31	1.34	1.482
	IKH-A/2	3	1.86	2.02	2.19	3	1.40	1.42	1.43	1.421
	TAT-E/3	2	1.92	1.94	1.95	2	1.28	1.35	1.41	1.439
	SHG-A/9	0	–	–	–	1	–	1.17	–	–
	TAT-C/7	1	–	2.00	–	1	–	1.39	–	1.440
	TAT-C/2	1	–	1.95	–	1	–	1.34	–	1.459
	TAT-D/1	1	–	1.77	–	1	–	1.30	–	1.367
m2	TAT-C/2	2	1.85	1.89	1.93	2	1.48	1.50	–	1.256
	SHG-A/20	2	1.80	1.89	1.98	2	1.44	1.56	1.68	1.213
	TGR-AB/22	2	1.75	1.83	1.91	2	–	1.56	1.60	–
	TGR-A/13	1	–	1.83	–	1	–	1.50	–	1.221
	TGR-B/1	3	1.86	1.91	1.94	3	–	1.53	1.57	1.254
	IKH-A/3-4	0	–	–	–	1	–	1.50	–	–
	IKH-A/2	2	1.86	1.91	1.96	3	1.44	1.52	1.58	1.256
	TAT-E/3	1	–	1.90	–	1	–	1.58	–	1.201
	SHG-AB/17-18	4	1.90	1.97	2.11	4	–	1.60	1.71	1.230
	TAT-C/7	2	–	1.91	1.97	2	–	1.49	1.53	1.279
	TAT-C/6	1	–	1.82	–	1	–	1.52	–	1.197
	TGR-AB/21	2	1.92	1.94	1.95	2	1.52	1.53	1.54	1.268
	TAT-D/1	2	1.61	1.73	1.85	2	1.43	–	1.51	–
m3	SHG-AB/17-18	6	1.77	1.83	1.98	6	1.37	1.48	1.58	1.236
	SHG-A/20	1	–	1.74	–	1	–	1.42	–	1.224
	TGR-AB/21	5	1.83	1.89	1.99	5	1.44	1.52	1.60	1.246
	TGR-B/1	0	–	–	–	1	–	1.45	–	–
	IKH-A/3-4	1	–	1.77	–	1	–	1.35	–	1.310
	IKH-A/2	1	–	1.96	–	1	–	1.54	–	1.273
	TGL-A/11c	1	–	1.95	–	1	–	1.61	–	1.216
	TAT-C/6	1	–	1.79	–	1	–	1.57	–	1.141
	TAT-C/2	2	1.57	1.61	1.66	2	1.39	1.39	1.40	1.157
	TAT-D/1	1	–	1.69	–	1	–	1.33	–	1.271
	***E. caducus***	***N***	**Min**	**Mean**	**Max**	***N***	**Min**	**Mean**	**Max**	***L***/***W***
M1	SHG-A/20	1	2.29	2.29	2.29	1	–	1.43	–	1.604
	SHG-A/15+20	1	–	2.12	–	2	1.37	1.38	1.39	1.535
	IKH-A/2	1	–	2.31	–	1	–	1.59	–	1.455
	DEL-B/7	1	–	2.12	–	1	–	1.52	–	1.390
	SHG-C/1	1	–	2.00	–	1	–	1.43	–	1.396
	TAT-C/3	1	–	1.89	–	1	–	1.25	–	1.509
	TAT-D/1	8	1.81	1.99	2.23	8	1.34	1.40	1.49	1.420
M2	TGR-ZO/2	2	1.61	1.61	1.61	2	1.40	1.40	1.40	1.152
	IKH-A/1	2	1.56	1.60	1.64	2	1.42	1.46	1.49	1.097
	TGL-A/11b	1	–	1.60	–	1	–	1.52	–	1.051
	SHG-C/1	1	–	1.69	–	1	–	1.48	–	1.138
	TAT-C/1	2	1.53	1.57	1.61	2	1.44	1.45	1.46	1.080
	TGL-A/2	3	1.50	1.56	1.62	3	1.50	1.56	1.38	1.000
	TGR-A/14	2	1.54	1.56	1.59	2	1.40	1.41	1.42	1.106
	TAT-D/1	5	1.41	1.50	1.60	6	1.36	1.41	1.46	1.069
M3	TGR-B/1	1	–	1.18	–	1	–	1.23	–	0.959
	IKH-A/1	1	–	1.33	–	1	–	1.33	–	1.000
	TGL-A/11b	1	–	1.29	–	1	–	1.29	–	1.000
	TAT-C/6	1	–	1.40	–	1	–	1.33	–	1.051
	TAT-D/1	2	1.23	1.24	1.24	3	1.24	1.26	1.28	0.982
m1	IKH-A/1	1	–	1.79	–	1	–	1.36	–	1.311
	TAT-E/3	2	1.45	1.62	1.80	2	1.07	1.15	1.23	1.415
	SHG-A/9	1	–	1.73	–	1	–	1.20	–	1.440
	TAT-C/7	1	–	1.59	–	1	–	1.07	–	1.491
	SHG-C/1	1	–	1.53	–	1	–	1.12	–	1.369
	TAT-C/2	1	–	1.68	–	1	–	1.15	–	1.464
	TGL-A/2	2	1.49	1.52	1.55	2	1.07	1.09	1.11	1.392
	TGR-A/14	0	–	–	–	1	–	1.11	–	–
	TAT-D/1	11	1.52	1.65	1.88	11	1.03	1.16	1.32	1.423
m2	UNCH-A/3	1	–	1.77	–	1	–	1.44	–	1.229
	SHG-A/15+20	1	–	1.76	–	1	–	1.42	–	1.236
	SHG-A/15	1	–	1.58	–	1	–	1.24	–	1.278
	TGR-AB/22	1	–	1.52	–	1	–	1.27	–	1.198
	TGR-AB/21	1	–	1.44	–	1	–	1.20	–	1.203
	TGR-ZO/1	1	–	1.54	–	1	–	1.26	–	1.226
	TGR-B/1	1	–	1.54	–	1	–	1.27	–	1.211
	TAT-E/3	1	–	1.43	–	1	–	1.26	–	1.135
	SHG-A/9	3	1.69	1.75	1.81	3	1.43	1.46	1.51	1.192
	TAT-C/7	1	–	1.43	–	1	–	1.20	–	1.190
	TAT-C/6	1	–	1.58	–	1	–	1.27	–	1.248
	SHG-C/1	2	1.55	1.59	1.63	2	–	1.31	–	1.216
	TGL-A/2	3	1.49	1.56	1.62	3	1.27	1.30	1.34	1.194
	TGR-A/14	1	–	1.36	–	1	–	1.12	–	1.216
	TGR-A/13	1	–	1.79	–	1	–	1.35	–	1.327
	TAT-D/1	11	1.41	1.60	1.77	11	1.14	1.32	1.40	1.209
m3	UNCH-A/3	1	–	1.75	–	0	–	–	–	–
	TGL-AB/22	1	–	1.60	–	1	–	1.31	–	1.227
	TAT-E/3	1	–	1.32	–	1	–	1.16	–	1.137
	TAT-C/3	1	–	1.45	–	1	–	1.26	–	1.148
	TGR-A/14	1	–	1.32	–	1	–	1.11	–	1.189
	TAT-D/1	5	1.31	1.49	1.66	5	1.15	1.27	1.34	1.175
	***E. bagus/E***. **cf** ***. bagus***	***N***	**Min**	**Mean**	**Max**	***N***	**Min**	**Mean**	**Max**	***L***/***W***
M1	TAT-E/27	1	–	1.88	–	1	–	1.30	–	1.448
	DEL-B/12	1	–	1.66	–	1	–	0.99	–	1.680
	TGW-A/2b	7	1.77	1.83	1.91	7	1.15	1.24	1.30	1.480
	TGW-A/2a	18	1.05	1.89	2.14	18	1.05	1.25	1.39	1.515
	TAR-A/2	4	–	1.66	–	4	–	1.21	–	1.372
	ABO-A/3	2	1.82	1.85	1.88	2	1.37	1.38	1.40	1.341
	TGR-C/2	6	1.66	1.89	2.07	6	1.08	1.21	1.32	1.556
	TGR-C/1	13	1.56	1.76	2.07	13	1.07	1.17	1.29	1.509
	IKH-A/1	0	–	–	–	1	1.37	1.37	1.37	–
M2	TGW-A/2b	11	1.19	1.34	1.49	11	1.00	1.18	1.31	1.142
	TGW-A/2a	9	1.22	1.37	1.43	9	1.04	1.19	1.26	1.151
	TAR-A/2	3	1.37	1.37	1.38	3	1.16	1.16	1.16	1.180
	ABO-A/3	0	–	–	–	1	–	1.24	–	–
	TGR-C/2	6	1.22	1.33	1.46	6	1.04	1.16	1.36	1.142
	TGR-C/1	13	1.20	1.33	1.39	13	1.02	1.13	1.22	1.174
	UNCH-A/3	1	1.43	1.43	1.43	1	1.33	1.33	1.33	1.074
	IKH-A/3-4	1	–	1.51	–	1	–	1.14	–	1.324
	TAT-E/3	1	–	1.59	–	0	–	–	–	–
M3	TGW-A/2b	5	0.94	0.98	1.03	5	1.01	1.04	1.07	0.941
	TGW-A/2a	5	0.95	1.02	1.12	5	0.94	1.04	1.12	0.977
	TAR-A/2	1	–	1.00	–	1	–	1.01	–	0.985
	TGR-C/2	1	–	0.91	–	1	–	0.95	–	0.956
	TGR-C/1	1	–	1.08	–	1	–	0.96	–	1.125
m1	TAT-surf	1	–	1.77	–	1	–	1.18	–	1.500
	DEL-B/12	1	–	1.61	–	1	–	1.09	–	1.478
	TGW-A/2b	7	1.33	1.45	1.68	7	0.92	1.00	1.07	1.454
	TGW-A/2a	7	1.45	1.56	1.63	8	0.98	1.07	1.16	1.452
	TAR-A/2	4	1.45	1.47	1.50	4	0.92	0.96	0.98	1.533
	TGR-C/1	13	1.40	1.44	1.56	13	0.92	0.99	1.06	1.450
	TGR-C/2	10	1.29	1.48	1.61	10	0.91	0.99	1.08	1.488
m2	TAT-surf	1	–	1.62	–	1	–	1.38	–	1.174
	IKH-B/5	1	–	1.24	–	1	–	1.05	–	1.181
	DEL-B/12	1	–	1.32	–	1	–	0.98	–	1.342
	TGW-A/2b	3	1.46	1.47	1.49	3	1.11	1.13	1.17	1.306
	TGW-A/2a	3	1.43	1.45	1.50	3	1.08	1.13	1.20	1.291
	TAR-A/2	0	–	–	–	1	–	1.07	–	–
	ABO-083	1	–	1.51	–	1	–	1.19	–	1.273
	ABO-A/3	1	–	1.55	–	1	–	1.29	–	1.206
	TGR-C/2	7	1.31	1.36	1.45	7	1.03	1.09	1.16	1.253
	TGR-C/1	15	1.27	1.41	1.55	15	1.03	1.09	1.18	1.293
m3	TAT-surf	1	1.31	1.31	1.31	1	1.19	1.19	1.19	1.101
	TGW-A/2b	3	1.38	1.46	1.60	3	1.10	1.16	1.27	1.254
	TGW-A/2a	2	1.51	1.56	1.61	3	1.22	1.26	1.32	1.242
	TGR-C/2	3	1.06	1.35	1.50	3	0.94	1.11	1.25	1.220
	TGR-C/1	1	–	1.02	–	2	0.92	1.02	1.11	1.005
	***E. jilantaiensis***	***N***	**Min**	**Mean**	**Max**	***N***	**Min**	**Mean**	**Max**	***L***/***W***
M1	TGW-A/2b	7	1.91	2.11	2.32	7	1.25	1.36	1.45	1.545
	TGW-A/2a	1	–	2.27	–	1	–	1.53	–	1.489
	TGW-A/1	1	–	2.40	–	1	–	1.47	–	1.631
M2	TGR-C/1	2	1.51	1.54	1.56	2	1.26	1.28	1.29	1.204
	TGW-A/2a	4	1.47	1.56	1.71	4	1.29	1.38	1.57	1.132
	TGW-A/2b	6	1.34	1.48	1.61	6	1.16	1.31	1.37	1.130
	TAR-A/2	1	–	1.56	–	0	–	1.48	–	1.054
M3	TGR-C/1	1	–	1.12	–	1	–	1.18	–	0.953
m1	TGR-C/1	1	–	1.77	–	1	–	1.13	–	1.563
	TGW-A/2a	12	1.73	1.84	1.97	11	1.06	1.18	1.28	1.561
	TGW-A/2b	4	1.84	1.90	1.96	4	1.15	1.20	1.27	1.586
m2	TAR-A/2	1	–	1.74	–	1	–	1.26	–	1.385
	TGR-C/1	3	1.60	1.62	1.65	3	1.23	1.27	1.30	1.276
	TGW-A/2a	6	1.56	1.66	1.76	8	1.23	1.28	1.32	1.297
	TGW-A/2b	5	1.51	1.61	1.67	5	1.18	1.21	1.27	1.327
m3	TGW-A/2b	3	1.26	1.31	1.40	3	1.04	1.14	1.23	1.148
	***E. occasionalis/E***. **cf** ***. occasionalis***	***N***	**Min**	**Mean**	**Max**	***N***	**Min**	**Mean**	**Max**	***L***/***W***
M1	IKH-A/2	1	–	1.71	–	1	–	1.14	–	1.500
	TGW-AB/22	1	–	1.68	–	1	–	1.13	–	1.377
M2	IKH-A/2	3	1.21	1.31	1.39	3	1.18	1.22	1.25	1.073
m2	IKH-A/2	1	–	1.57	–	1	–	1.23	–	1.276
m3	TGW-AB/22	1	–	1.24	–	1	–	1.03	–	1.204

Measurements are in millimeter

*Min* minimum value, *Max* maximum value, *N* number of specimens, *L/W *length-Width ratio

## Systematic palaeontology

Order Rodentia Bowdich, 1821

Superfamily Muroidea Illiger, 1811

Family Cricetidae Brandt, 1855

Subfamily Eucricetodontinae Mein and Freudenthal, 1971

Genus *Eucricetodon* Thaler, 1966

*Eucricetodon asiaticus* (Matthew and Granger, 1923)

Fig. 3

**Fig. 3:**
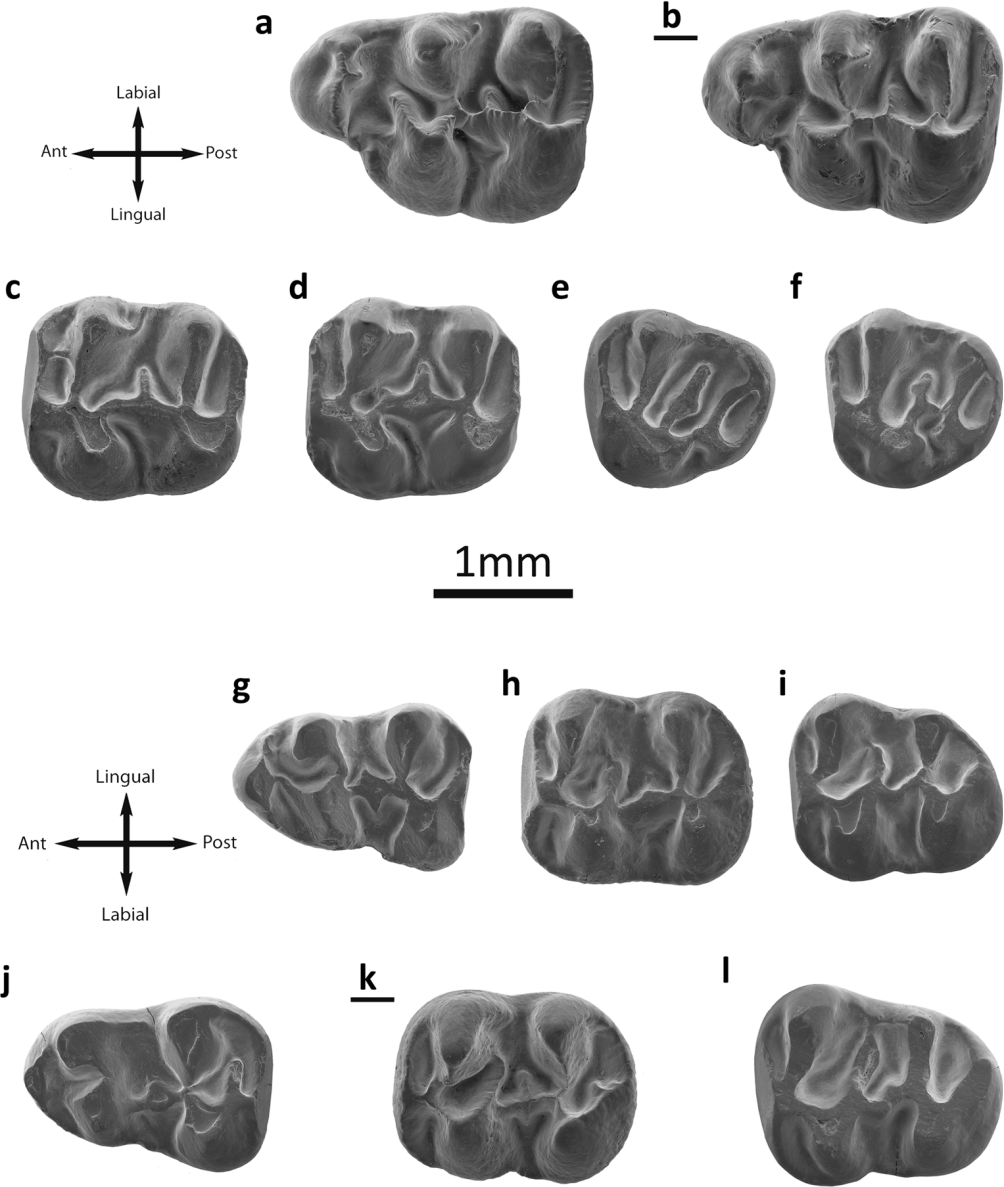
*Eucricetodon asiaticus* from the Valley of Lakes. **a** Taatsiin Gol right locality, fossil layer TGR-AB/22, inverted right M1 (NHMW2015/0257/0002). **b** Tatal Gol locality, fossil layer TAT-C/3, left M1 (NHMW2015/0249/0001). **c** Hsanda Gol locality, fossil layer SHG-A/20, left M2 (NHMW2015/0243/0001). **d** Fossil layer SHG-AB/17-18, left M2 (NHMW2015/0245/0004). **e** Fossil layer SHG-A/20, left M3 (NHMW2015/0243/0003). **f** Fossil layer SHG-AB/17-18, left M3 (NHMW2015/0245/0007). **g** Ikh Argalatyn Nuruu locality fossil layer IKH-A/3-4, left m1 (NHMW2015/0241/0002). **h** Tatal Gol locality, fossil layer TAT-D1, left m1 (NHMW2015/0252/0003). **i** Fossil layer TAT-C/2, left m3 (NHMW2015/0248/0006). **j** Fossil layer TAT-C/2, left m1 (NHMW2015/0248/0002). **k** Fossil layer TAT-C/2, inverted right m1 (NHMW 2015/0248/0004). **l** Ikh Argalatyn Nuruu locality, fossil layer IKH-A/2, left m3 (NHMW2015/0240/0010)

Synonymy

2014 *Eucricetodon* aff. *asiaticus*—Maridet et al. Table 3. p. 264. (Only for the localities: TAT-D/1; TGR-A13; TGR-A/14; TAT-C1-3; SHG-C1-2; TAT-C/6+7; SHG-A6-9; TGL-A/11; TAT-E/3; IKH-A/1; TGR-B/1; TGR-AB/21; TGR-AB/22; SHG-AB/15-20; SHG-AB/17-18; and UNCH-A3B)

**Original type locality:** Hsanda Gol Formation, near Loh, Mongolia

**Stratigraphic range:** Early Oligocene (local biozones A and B)

**Geographical range:** Central Asia

**Material:** See Table 1 (catalogue numbers NHMW2009z/0135/0001-18; NHMW2009z/0142/0001-19; NHMW2015/0239-245; NHMW2015/0247-258)

**Measurements:** Given in Table 2

Description

**M1** (27 specimens): The enamel is thick. The molar has three roots, two on the labial part and a wider one on the lingual side. Its crown is high: the cusps are stout and rounded. The prelobe is present in only one specimen (SHG-C/1). The anterocone is mostly simple and wide, but it can be rounded (IKH-A/3-4; SHG-AB/17-18) or even slightly split (TGR-AB/17-18, 21, 22). The anterocingulum is present in only one case (SHG-C/1). The anterocone spur is usually present (Fig. 3a, b) is long, connected to the paracone in one tooth (TGR-B/1) and it is absent in two (TGR-AB/21). The lingual anteroloph is absent in only two specimens (IKH-A/3-4, SHG-AB/17-18). There is no anterolabial style or cingulum. The labial anteroloph is always present (Fig. 3a, b). The anterolophule is mostly present, only one molar (TAT-C/3) lacks of it; it does not reach the protocone in one case (SHG-AB/17-18). The protolophule I is present in all teeth (Fig. 3a, b) but one (TAT-C/1) and all the cases have protolophule II. Four molars have a platform on the protosinus (IKH-A/3-4; TGR-B/1; TGR-AB/22). The protostyle is frequently present (Fig. 3a); seven of them have a spur. The anterior arm of the protocone is mostly present and short (only absent in SHG-C/1); it is connected to the anterocone in three specimens (SHG-AB/17-18; TGR-AB/21, 22). The paracone is always rounded; it has a spur in most of the cases (Fig. 3a, b; it is absent in IKH-A/3-4 and SHG-AB/17-18). In one case, it is curved (TAT-C/3). The mesosinus is closed in all fossils but one (TGR-AB/21). The mesostyle is always absent. The mesoloph is present in almost all cases (absent in TAT-C/2); it is well developed; short in three specimens (TAT-C/3, TGR-B/1, TGR-AB/21, and TGR-AB/22). Only one tooth (SHG-C/1) displays a second mesoloph. The metalophule is connected to the anterior arm of the hypocone (TAT-C/2, C/3, TGR-B/1, and TGR-AB/21), transversal (SHG-C/1, SHG-AB/17-18, TGR-AB/22) or joined to the posterior arm of the hypocone (IKH-A/3-4, SHG-AB/17-18). The posteroloph is long and reaches the metacone. The sinus is usually transversal; it is retroverse in some specimens (IKH-A/3-4, SHG-AB/17-18, TGR-AB/22). The lingual cingulum can be present or absent (Fig. 3a, b).

**M2** (27 specimens): The enamel is thick (Fig. 3c, d). Both lingual and labial anterolophs are well developed, but the labial one is weak in some specimens. The protolophule I is connected to the anterolophule (Fig. 3c, d); it is disconnected in one molar (SHG-AB/17-18). One case (SHG-A/20) present protolophule spur. The second mesoloph is present (Fig. 3d); it can be short or long, and in some teeth (UNCH-A/3), it is connected to the paracone. The mesoloph is present and its length is about the half of the mesosinus length. The entomesoloph is absent. The paracone spur is present; it can be weak or curved (Fig. 3c) and well-developed reaching the mesosinus (Fig. 3c). The metalophule is connected to the anterior part of the hypocone. When the tooth presents strong wear, it seems to be connected to the middle part of the hypocone. The posteroloph is well-developed and long. The sinus is strongly proverse; it is closed by a small lingual cingulum.

**M3** (11 specimens): Both lingual and labial anterolophs are present, but the lingual is less developed than the labial, in some specimens is difficult to distinguish (Fig. 3e). The protolophule I is present and connected to the anterolophule; protolophule II is absent. The entoloph is transversally oriented and is connected to the middle part of the protocone (Fig. 3f). The mesoloph is always present and it is well developed; it is placed in the middle part of the entoloph (Fig. 3e, f). The posterior part of the entoloph is longitudinally oriented, and it is connected to the metalophule. In some specimens (SHG-A/20), this posterior part of the entoloph is missing. The neoentoloph is present in most of the molars (Fig. 3e); it is present but disconnected in one fossil (SHG-AB/17-18). Thus, the sinus is short and transversal. The hypocone is extremely reduced (Fig. 3e, f). The metalophule is connected to the anterior arm of the hypocone and the neoentoloph. The posteroloph is always present and long. The mesosinus is closed by a cingulum and in some molars (SHG-AB/17-18) a small mesostyle is also present. The sinus is closed by a small cingulum.

**m1** (28 specimens): This molar has an elongated shape. The anteroconid is situated on the longitudinal axis of the occlusal surface; it is transversally elongated. The labial anterolophid is a well-developed ridge that connects the anteroconid with the protoconid (Fig. 3g, j). The lingual anterolophid is present but it is shorter than the labial one; it can reach the metaconid in some cases (Fig. 3g; TGR-AB/21). The anterolophulid is present and connected to the middle part of the anteroconid (Fig. 3g, j). In one molar (TGR-AB/21), it is not present. The metalophulid I is missing and metalophulid II is always present and connected to the posterior arm of the protoconid. In one case (TGR-B/1), there is no metalophulid and the posterior arm of the protoconid ends freely in the mesosinusid. The ectolophid bears a mesolophid. This mesolophid is usually well-developed but it does not reach the lingual border; it can also be short (Fig. 2g, j). The ectomesolophid is usually present but short or incipient. The entoconid spur is present in some specimens (TAT-E/3; TGR-AB/22). The hypoconid hind arm is present in some cases (Fig. 3j; SHG-AB/17-18; TGR-B/1; TGR-AB/21; TAT-E/3; IKH-A/2). The hypolophulid is short and connected to the ectolophid. The sinusid is short and wide, transversally directed.

**m2** (28 specimens): Both labial and lingual anterolophids are present and well developed (Fig. 3k). The metalophulid I is present, and it is connected to the anterolophulid. The metalophulid II is absent. The posterior arm of the protoconid is well-developed and long; it usually ends freely in the mesosinusid, but it is curved (Fig. 3h) and connected to the metaconid sometimes (Fig. 3k; TGR-AB/21; TGR-AB/22; IKH-A/2). The mesolophid is present in some specimens (SHG-A715; TAT-C/7; TGR-AB/21; TGR-AB/22; TAT-E/3; IKH-A/3), and it is always short (Fig. 3k). In most teeth, the ectomesolophid is present but is short or incipient (Fig. 3h, k). The ectolophid is oblique. The sinus is wide and transversal, a cingulum is connecting the hypoconid with the protoconid at the labial border of the sinusid. The hypolophulid is connected to the posterior part of the ectolophid. The hypoconid hind arm is present in some cases (Fig. 3k; TGR-AB/21; TGR-AB/22; TAT-E/3; TAT-C/6).

**m3** (31 specimens): The labial anterolophid is long and it reaches the protoconid. The lingual anterolophid is also long and connected to the metaconid (Fig. 3i, l). The metalophulid I is present and it is connected to the anterolophulid. Two specimens have a metalophulid spur (TGR-AB/21). The metalophulid II is absent. The posterior arm of the protoconid is well developed and longer than in the m2 (Fig. 3i, l); it ends freely in the mesosinusid (Fig. 3i) or reaches the lingual border. In most cases, it is connected to a cingulid present in the mesosinusid (Fig. 3l), but it is joined to the entoconid in one molar (TGR-AB/21) and to the metaconid in another (TGR-AB/21). The mesolophid is present in a few fossils (IKH-A/2; TGR-B/1; TGR-AB/21; TGR-AB/22) but it is always weak. The ectomesolophid is always present but short (Fig. 3i). The hypolophulid is connected to the anterior part of the hypoconid (Fig. 3i). One molar displays a posterior hypoconid arm (TGR-AB/21).

**Remarks:** The studied material presents bunodont teeth; single anteroconid and hypoconid hind arm which are common in *Eucricetodon* (Li et al. 2016). It has also a number of features that fit the emended diagnosis for *E. asiaticus* made by Gomes Rodrigues et al. (2012a): the M1 with a simple anterocone and an anterior arm of the protocone usually free, a metalophule joining the mesial or middle part of the hypocone. The m1 have an anteroconid developed, central, isolated, or linked to the protoconid. The Mongolian material follows in general the trends described for *Eucricetodon* by Li et al. (2016). Single anterocone and well-developed anterocone spur is considered as basal trait (Li et al. 2016); the anterocone is split on the younger localities from the biozone B. The anterocone spur is more developed on the older sites (TGR-B/1) than on the younger and can even be absent (TGR-AB/21). The single protocone–paracone connection is also a basal feature (Li et al. 2016) and is the general condition in the whole sample. An anterior connection of the metalophule is also basal (Maridet et al. 2009; Gomes Rodrigues et al. 2012a; Li et al. 2016). The Mongolian material displays posterior connection on the younger localites. As a rule, the studied material shows weak ectomesolophid which is considered as basal (Li et al. 2016). To sum up, *E. asiaticus* from Mongolia presents a general basal morphology and follows the trends described for the genus.

*E. asiaticus* is found in the Oligocene of Asia. It was first described by Matthew and Granger (1923) from the sediments of Hsanda Gol Formation (Mongolia), but detailed descriptions of the type material were provided by Lindsay (1978). *E. asiaticus* have been recovered from other Asian localities. Gomes Rodrigues et al. (2012a) described several specimens from Ulantatal area of Nei Mongol (UTL 1 (Ulan I), 3, 4, 5, 7 (Ulan II), 8 (Ulan III), late early Oligocene to late Oligocene). The Mongolian fossils and *E. asiaticus* from Ulantatal share the presence of a weak paracone spur; an anterocone spur and anterolophule (sensu Gomes Rodrigues et al. 2012a) on the M1. Besides, the M2s from both areas have second mesoloph and the m2s display a long posterior arm of the protoconid. Also, the size of the Mongolian material is similar to those fossils from Ulantatal collections and the *L*/*W* ratio is the same for both assemblages (Table 2). The younger localities from Ulantatal do not present a clearly advanced morphology according to the trends described for *Eucricetodon* by Li et al. (2016).

*Eucricetodon caducus* (Shervyreva, 1967)

Fig. 4

**Fig. 4:**
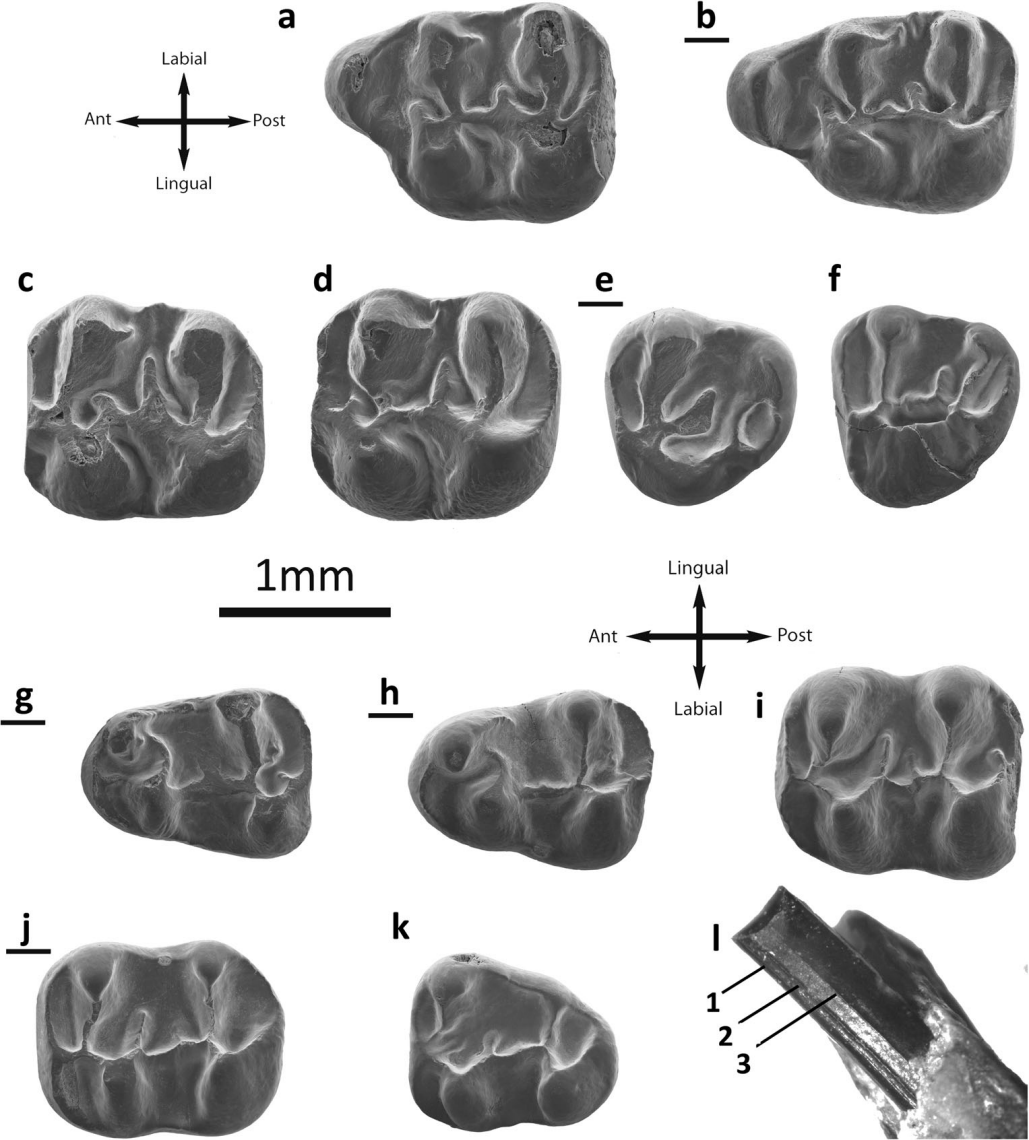
*Eucricetodon caducus* from the Valley of Lakes. **a** Taatsiin Gol Right locality, fossil layer TGR-AB/22, inverted right M1 (NHMW2015/0287/0007). **b** Tatal Gol locality, fossil layer TAT-D/1, left M1 (NHMW2015/0284/0001). **c** Taatsiin Gol Left locality fossil layer TGL-A/2, left M2 (NHMW2015/0290/0002). **d** Tatal Gol locality, fossil layer TAT-C/1, left M2 (NHMW2015/0282/0002). **e** fossil layer TAT-D/1, inverted right M3 (NHMW2015/0287/0013). **f** Taatsiin Gol Right locality, fossil layer TGR-B/1, left M3 (NHMW2015/0295/0002). **g** Taatsiin Gol left locality fossil layer TGL-A/2, inverted right m1 (NHMW2015/0290/0006). **h** Tatal Gol locality, fossil layer TAT-D1, left m1 (NHMW2015/0287/0019). **i** Taatsiin Gol Left locality fossil layer TGL-A/2, left m2 (NHMW2015/0290/0008). **j** Hsanda Gol locality, fossil layer SHG-A/15, inverted right m2 (NHMW2015/0278/0001). **k** Taatsiin Gol Right locality fossil layer TGR-A/14, left m3 (NHMW2015/0292/0006) **l** Tatal Gol locality, fossil layer TAT-D1, right incisor (NHMW2015/0287/0015)

Synonymy

2014 *Eucricetodon* aff. *bagus*—Maridet et al. Table 3. p. 264. (Only for the localities: DEL-B/7; IKH-A/1; SHG-A/15+20; SHG-A/20; SHG-A/9; TAT-E/3; TGL-A/11b; TGR-AB/22; TGR-B/1; TGR-ZO/1; TGR-ZO/2; UNCH-A/3)

2014 *Eucricetodon asiaticus*—Maridet et al. Table 3. p. 264. (Only for the localities: TAT-C/2 *pro parte*; TAT-C/6 *pro parte*; TGR-A/13 *pro parte*)

2014 *Eocricetodon* cf. *meridionalis*—Maridet et al. Table 3. p. 264. (Only for the localities: SHG-A/15; TAT-D/1; TAT-E/3 *pro parte*; TGR-A/14; TGR-AB/22)

**Original type locality:** Akespe, Aral Formation, Kazakhstan (early Oligocene)

**Stratigraphic range:** Early Oligocene (local biozones A and B)

**Geographical range:** Central Asia

**Material:** See Table 1 (catalogue numbers NHMW2009z/0132/0001-6; NHMW2015/0275-298)

**Measurements:** Given in Table 2

Description

**Incisor:** The buccal surface of the lower incisor presents three parallel lines and there is no oblique ornamentation.

**M1** (25 specimens): The cusps are stout and rounded. The anterocone is anteriorly enlarged and clearly simple; it can be rounded on the apex or can be labial-lingually elongated (DEL-B/7). It is situated on the labial side of the occlusal surface. There is no prelobe nor anterocingulum. The labial and lingual anterolophs are well developed and long; they reach the paracone and protocone, respectively. The anterocone spur is present in some specimens; it is long and connected to the anterior arm of the procotone in some molars (SHG-C/1; SHG-A/20). The anterolophule is missing; it can be mistaken for the anterior arm of the protocone when is connected to the spur of the anterocone. The protolophule I is always absent. The protolophule II is always present, and it is joined to the entoloph. Some teeth display a protostyle (IKH-A/2; SHG-C/1; TGR-A/13) with a protostyle spur that ends on the protocone. The posterior arm of the protocone ends freely in the sinus and the entoloph is attached to the middle part of the protocone. In some cases (TGR-A/14), the posterior arm of the protocone is connected to the entoloph. The posterior spur of the paracone is always present, but an anterior spur is present as well (TAT-D/1). All teeth display a short mesoloph. The second mesoloph is present in some specimens (Fig. 4b) but weak. The metalophule is proverse, connected to the anterior arm of the hypocone. In those fossils with strong wear, it is more central. The labial posteroloph is long. The sinus is proverse in those in which the posterior arm ends freely; it is straight on the rest.

**M2** (20 specimens): Both lingual and labial anterolophs are well developed. The protolophule I, present in all but one (Fig. 4d), is connected to the anterolophule. The protophule II is present in three molars (TAT-D/1; Fig. 4c). In some specimens, a labial spur on the anterolophule is observed (Fig. 4c). The second mesoloph is present in several molars. The mesoloph is present and its length is about the half of the mesosinus length or longer (TGL-A/2a) reaching the labial border. The mesosinus is closed by a labial cingulum. The entomesoloph is absent. The paracone bears a spur; it is usually weak, but it can be longer, curved, and reaching the mesosinus (TGR-A/14; Fig. 4d). The metalophule is connected to the entoloph, clearly anterior to the hypocone. The posteroloph is well developed and long. The sinus is strongly proverse; it is open.

**M3** (11 specimens): The labial anteroloph is present and long whereas the lingual one is weak and in some specimens is absent (TAT-D/1). The protolophule I is present and connected to the anterolophule. The protolophule II is absent. The entoloph is present; it can be curved and connected to the middle part of the protocone or connected to the protolophule (Fig. 4f); it can also be incomplete (Fig. 4f). The mesoloph is always present; it is usually well developed and can reach the labial border. The posterior part of the entoloph is longitudinally oriented, and it is connected to the metalophule but in one specimen is missing (Fig. 4e). The neoentoloph is always present and continuous. The sinus is short and transversal. The hypocone is extremely reduced. The metalophule is connected to the anterior arm of the hypocone. The posteroloph is always present and long. The mesosinus is closed by a cingulum, and in some fossils (SHG-AB/17-18), a small mesostyle is also present.

**m1** (23 specimens): This molar has an elongated shape. The anteroconid is situated on the longitudinal axis of the occlusal surface; it is transversally elongated and simple. The labial anterolophid is a well-developed ridge that connects the anteroconid with the labial part of the protoconid. The lingual anterolophid is present and reaches the metaconid in its anterior part. The anterolophulid is present in some molars and connected to the middle part of the anteroconid (Fig. 4g; TAT-D/1) but it is mostly absent (Fig. 4h). The metalophulid I is missing. The metalophulid II is always present and connected to the posterior arm of the protoconid. The ectolophid is joined to the base of the protoconid. The mesolophid is frequently present; it can be weak (Fig. 4g) or developed, but it never reaches the lingual border. The ectomesolophid is usually present, is short, or is incipient. The entoconid spur is present in all cases but two (Fig. 4g, h); it can be short or well developed (TAT-C/2). The hypolophulid is long and connected to the ectolophid. The hypoconid hind arm is always present. The mesosinusid is wide and can be open (Fig. 4g) or closed by a cingulid. The sinusid is short and wide, transversally directed, and closed by a cingulid.

**m2** (34 specimens): Both labial and lingual anterolophids are present and well developed. The metalophulid I is present and is connected to the anterolophulid. The metalophulid II is absent. The posterior arm of the protoconid is connected to the ectolophid. Most of the cases present a prolongation of the protoconid hind arm that can reach the metaconid (TAT-D/1). The mesolophid is always present and it is short. The ectomesolophid is not present, but some specimens have an enlargement of the ectolophid in its labial part (TAT-D/1; TGL-A/2a). The ectolophid is horizontal. The sinusid is wide, transversal, and open. The mesostlylid is present in some cases (Fig. 4j). The hypolophulid is connected to the posterior part of the ectolophid. The hypoconid hind arm is present in some molars (Fig. 4i). The posterolophid is long and it displays a constriction (Fig. 4i).

**m3** (10 specimens): The labial anterolophid is long and it reaches the protoconid. The lingual anterolophid is also long and connected to the metaconid. The metalophulid I is present and it is connected to the anterolophulid. In one case, it is connected to the lingual anterolophid. The metalophulid II is absent, but one molar (TAT-D/1) presents a spur on the metalophulid I that is connected to the ectolophid. The ectolophid is long and thin; it bears a mesolophid in some fossils (Fig. 4k). The ectomesolophid is always absent. The entoconid is reduced and small. The hypolophulid is attached to the anterior part of the hypoconid. The hypoconid hind arm is absent.

**Remarks:** This material belongs also to *Eucricetodon* because it has hypoconid hind arm and single anterocone on the M1. As the above-described *E. asiaticus*, it is a big species (Table 2). However, it differs from *E. asiaticus* by its less-developed lingual anteroloph and its more-developed neoentoloph on the M3; its lingual anterolophid always present wider mesosinusids, longer hypolophulids, anterolophulid mostly absent and its longer ectomesolophid on the m1. In addition, the hypoconid hind arm is more frequent in *E. caducus*. The m2s possess always a mesolophid and a mesostylid. Moreover, the m3s lack a metalophulid spur or a hypoconid hind arm and show no ectomesolophid.

*E. caducus* was described by Shevyreva (1967) from the early Oligocene of Kazakhstan. The original descriptions were vague, and Wang (1987) emended the diagnosis based on Inner Mongolian fossils. The fossils here studied display the following diagnostic characters: presence of three parallel lines on the incisor enamel, short mesolophs, and metalophule joined to the anterior arm of the hypocone. *E. caducus* was previously recognised in the Mongolian sediments (Daxner-Höck et al. 2010; Maridet et al. 2014) and in the Wulanbulage Formation in China (Wang 1987). However, it is not found in Ulantatal area (Gomes Rodrigues et al. 2012a). There is a close species found in the late Oligocene from the Junggar basin (China) termed *Eucricetodon* aff. *caducus* that differs from the Mongolian material in having two longitudinal lines on the buccal surface of the incisors and faint oblique lines on the lateral face of some incisors, which is rare among *Eucricetodon* species. Also, the anterocone is always simple. The morphology of the M2 is simpler in *E. caducus* than *Eucricetodon* aff. *caducus* and the protolophule spur is not present. Also, the metalophulid II is not present in *E. caducus* from Mongolia.

*Eucricetodon bagus* Gomes Rodrigues et al., 2012a

Fig. 5

**Fig. 5:**
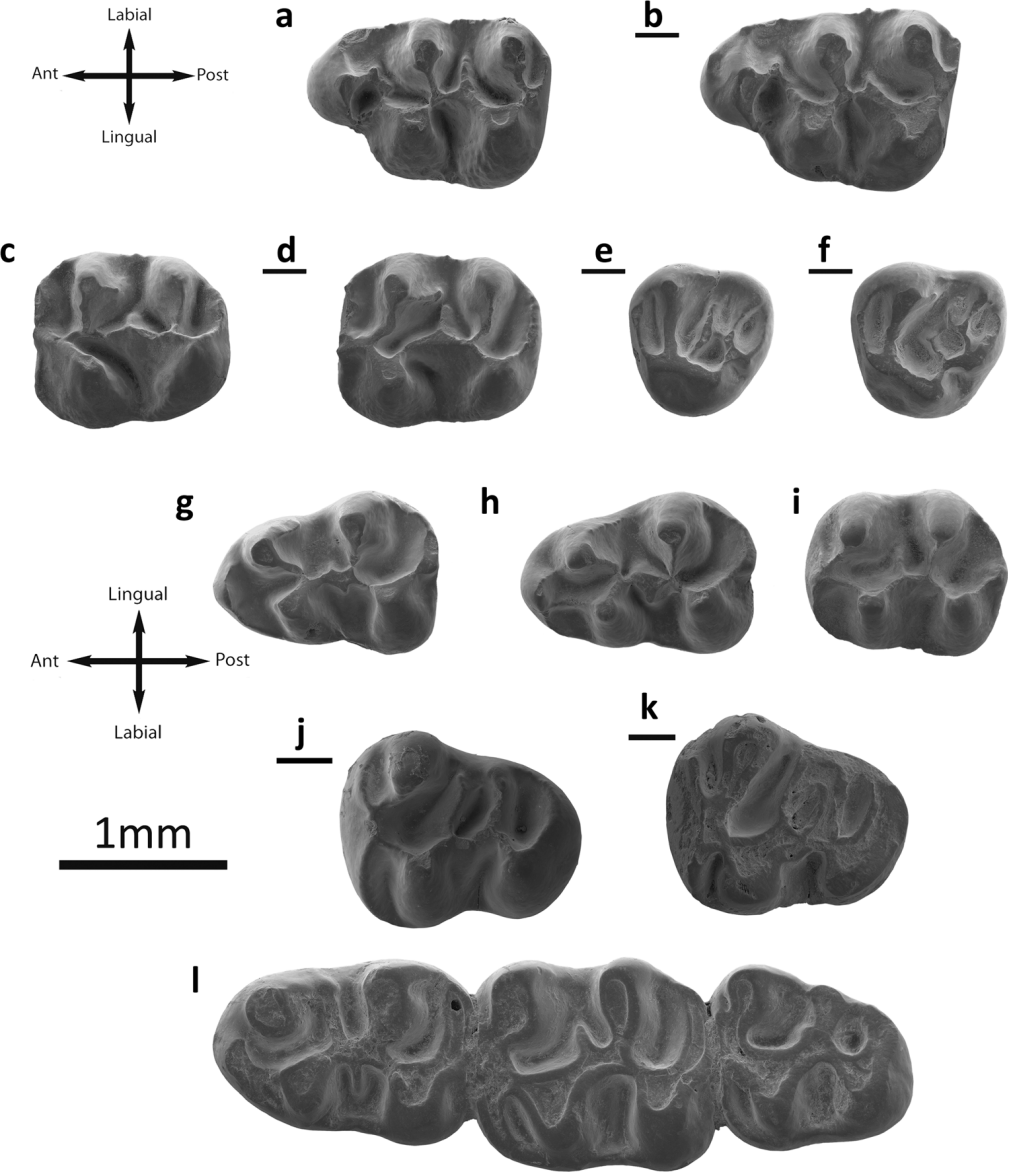
*Eucricetodon bagus* from the Valley of Lakes. **a** Unzing Khurem locality, fossil layer TAR-A/2, left M1 (NHMW2015/0266/0003). **b** Taatsiin Gol Right locality, fossil layer TGR-C/2, inverted right M1 (NHMW2015/0271/0004). **c** Left M2 (NHMW2015/0271/0011). **d** Inverted right M2 (NHMW2015/0271/0008). **e** Toglorhoi locality, fossil layer TGW-A/2b, inverted right M3 (NHMW2015/0273/0021). **f** Inverted right M3 (NHMW2015/0273/0022). **g** Fossil layer TGW-A/2a, left m1 (NHMW2015/0272/0038). **h** Left m1 (NHMW2015/0272/0034). **i** Taatsiin Gol Rigth locality fossil layer TGR-C/2, left m2 (NHMW2015/0271/0026). **j** Toglorhoi locality, fossil layer TGW-A/2a, inverted right m3 (NHMW2015/0273/0035). **k** Inverted right m3 (NHMW2015/0272/0045). *Eucricetodon* cf. *bagus*. **l** Tatal Gol locality, fossil layer TAT-surf, left mandible m1–m3 (NHMW2015/0246/0001)

Synonymy

2014 *Eucricetodon* aff. *caducus*—Maridet et al. Table 3. p. 264. (Only for DEL-B/12 and TAT-E/27)

2014 *Eucricetodon* aff. *bagus*—Maridet et al. Table 3. p. 264. (Only for IKH-A/1)

2014 *Eucricetodon* sp. 1—Maridet et al. Table 3. p. 264

2014 *Eucricetodon* sp. 2—Maridet et al. Table 3. p. 264

2014 *Eocricetodon* cf. *meridionalis*—Maridet et al. Table 3. p. 264. (Only for IKH-A/3-4 *pro parte*)

**Original type locality:** UTL4 (Ulan II), early late Oligocene, Ulantatal, Inner Mongolia, China

**New localities:** See Table 1

**Stratigraphic range:** Early-late Oligocene (local biozones B, C, and C1)

**Geographical range:** Central Asia

**Material:** See Table 1 (catalogue numbers *Eucricetodon bagus* NHMW2015/0260-262; NHMW2015/0264-274. *Eucricetodon* cf. *bagus* NHMW2015/0259/0001; NHMW2015/0263/0001-2; NHMW2015/0246/0001)

**Measurements:** Given in Table 2

Description

**M1** (nine specimens): The anterocone is rounded and large; it is usually simple, but it can be slightly split. A labial anterocingulum sometimes present (Fig. 4a) is fused with a cingulum on the anterosinus. The lingual anteroloph is well developed and departs from the apex of the anterocone towards the protocone and joins it. The anterocone spur is always present; it is long and connected to the anterior arm of the procotone (Fig. 5a, b) or ends freely in the anterosinus. The anterolophule is present and it is connected to the lingual anteroloph (Fig. 5a, b) in some cases and to the lingual part of the anterocone in others. The protostyle is rare and weak, only one molar displays it (TGR-C/2). The protolophule I is missing. The protolophule II is always present; it is thin and joined to the entoloph. The posterior spur of the paracone is always present, but in some specimens (TAT-D/1), an anterior spur is very weak and short. All teeth have short mesoloph. The second mesoloph is absent. The metalophule is posteriorly directed and is connected to the posterior arm of the hypocone or to the posteroloph. The labial posteroloph is long. The anterosinus and mesosinus are closed by a thick cingulum. The sinus can be straight or slightly proverse and closed by a cingulum.

**M2** (12 specimens): Both lingual and labial anterolophs are well developed. The protolophule I is usually present (TAR-A/2; TGR-C/1; ABO-A/3) and curved forward (Fig. 5c); it is connected to the point where the anterolophule is joined to the anterior arm of the protocone or is absent. The protolophule II is present in some molars (IKH-A/3; TGR-C/1; TGR-C/2; TAT-D/1; TGW-A/2a-/b; Fig. 5c). In one case, both protolophules are present (TGW-A/2a). The protolophule spur is displayed in some specimens (TGR-C/2; TGW-A/2b). The entoloph is straight and long; it bears a mesoloph that can be developed but it never reaches the labial border. The second mesoloph is not present. The paracone spur is always present; it is connected to the mesoloph in some cases (TAR-A/2). The entomesoloph is absent. The metalophule is usually connected to the entoloph, clearly anterior to the hypocone (Fig. 5c, d). In some molars, it is joined to the posterior arm of the hypocone (Fig. 5c; TGW-A/2a). The mesosinus is closed by a labial cingulum or by a style (TAR-A/2; TGR-C/1). The sinus is always proverse and closed by a small cingulum.

**M3** (one specimen): The labial anteroloph is present and long, and the lingual is absent. The protolophule I is present and connected to the short anterolophule. The protolophule II is absent. Some molars have a small paracone spur (Fig. 5f). The anterior part of the entoloph is incomplete, and some teeth display a small spur attached to the mesoloph (Fig. 5f). The posterior arm of the protocone is connected to the mesoloph, which is long but never reaches the labial border. The posterior part of the entoloph is present and joined to the metalophule. The neoentoloph is usually not present, only in a few specimens (Fig. 5e, f). The sinus is short and transversal. The hypocone is extremely reduced. The metalophule is connected to the point where the anterior arm of the hypocone and the entoloph are connected. The posteroloph is always present and long. The mesosinus is closed by a cingulum and in some fossils.

**m1** (17 specimens): This molar has an elongated shape. The anteroconid is situated on the longitudinal axis of the occlusal surface; it is transversally elongated and simple. The labial anterolophid is a well-developed ridge that connects the anteroconid with the labial part of the protoconid. The lingual anterolophid is missing or very short. The anterolophulid is present in some molars and connected to the labial part of the anteroconid (Fig. 5h; TAT-D/1); it may be absent (Fig. 5g) or join the metaconid instead of the protoconid. The metalophulid I is present in some specimens (Fig. 5h; TGR-C/2; TGW-A/2a-/2b). The metalophulid II is frequently present and connected to the posterior arm of the protoconid. In some cases neither metalophulid (I or II) is present (Fig. 5h; DEL-B/12; TAR-A/2). The ectolophid is joined to the posterior arm of the protoconid. The mesolophid is weak (Fig. 4g) or absent; it is long in one specimen (Fig. 5l). The ectomesolophid is always present and is weak but distinguishable. The entoconid spur is never present. The hypolophulid is short and connected to the ectolophid. The hypoconid hind arm is always absent. The mesosinusid is wide and open. The sinusid is short and wide, transversal, and either open or closed by a small cingulid (Fig. 5l).

**m2** (20 specimens): The molars are squared. Both labial and lingual anterolophids are present but the lingual one is weaker than the labial. The metalophulid I is present and it is connected to the point where the labial anterolophid and the anterolophulid are joined. The metalophulid II is always absent. The posterior arm of the protoconid is connected to the ectolophid. The protoconid hind arm is not prolonged. The mesolophid is always present and it is short. The ectomesolophid is absent. The ectolophid is horizontal. The sinusid is wide, transversal, and is closed by a small cingulid in some cases (Fig. 5l). The mesosinusid is usually open, but some specimens display a small cingulid closing it. The hypolophulid is transversal and connected to the posterior part of the ectolophid. The hypoconid hind arm is absent. The posterolophid is long and it displays a constriction (Fig. 5i).

**m3** (three specimens): The labial anterolophid is long and it reaches the protoconid. The lingual anterolophid is shorter and it does not reach the metaconid. The metalophulid I is present and is connected either to the anterior part of the anterior arm of the protoconid or to the lingual anterolophid (Fig. 5j, k). The metalophulid spur is absent (Fig. 5j, k). The metalophulid II is always absent. The ectolophid bears a long mesolophid that usually reaches the lingual border (Fig. 5j, k) or it is curved and attached to the entoconid. Some molars have a very weak ectomesolophid (Fig. 5j, k). The small entoconid is reduced, the hypoconid hind arm is absent, and the transverse hypolophulid is connected to the entolophid (Fig. 5j, k).

**Remarks:** The morphology and size of the studied material fit with the diagnosis of *E. bagus* described in Ulantatal by Gomes Rodrigues et al. (2012a). M1 has mostly a simple anterocone and frequently double connection between anterocone and protocone via the anterolophule and the anterior arm of the protocone. The M2 displays a style in the mesosinus and both protolophules. The m1 anteroconid is frequently isolated. Apart from that, we have found several similarities after the direct comparison with the holotype and the type material: the anterocone is split in some specimens; the anterocone spur is well developed; the paracone spur is present; the anterior arm of the protocone can be connected to the lingual anterolophid; and the metalophule is posteriorly directed on the M1. The anterior part of the entoloph is missing and the mesoloph displays a small spur on some M3. The m1 presents both metalophulids in some cases, and the m2 is well-developed anteriorly with a small anteroconid. The Mongolian sample presents low variability in size (Table 2). However, the slightly bigger size of the molars from the mandible NHMW-2015/0246/0001 is remarkable. The m1 displays a longer mesolophid than the other m1s, but given the scarcity of material in the locality, it is classified as *Eucricetodon* cf. *bagus*. The few specimens from IKH-A/3-4 and ABO-083, which are not characteristic elements, are also considered *Eucricetodon* cf. *bagus*. In comparison with the other species of *Eucricetodon* above described, a number of differences can be seen. It is more hypsodont than *E. caducus* and *E. asiaticus*, and the valleys are deeper and narrower; it does not present a second mesoloph. The metalophule is posteriorly directed and is connected to the posterior arm of the hypocone or to the posteroloph on the upper molars. The sinus is always proverse and closed by a small cingulum on the upper molars, whereas in *E. caducus* it is open. There is no lingual anteroloph on the M3. It has no lingual anterolophid, whereas *E. caducus* has it; the anterolophulid is connected to the labial part of the anteroconid. The metalophulid I is present in some m1s, and the ectolophid is connected to the posterior arm of the protoconid on the lower molars. It lacks hypoconid hind arm on the m1 (and m2). The lingual anterolophid is less developed than in *E. caducus*; the protoconid hind arm is not prolonged as *E. asiaticus* and *E. caducus*. In general, the cingular formations are not as developed as in *E. caducus* or *E. asiaticus* on the m2. The lingual anterolophid on the m3 is shorter than in *E. caducus*. The metalophulid I is lingually connected, and the mesolophid is more developed than is *E. caducus* on the m3.

*E. bagus* possesses in general derived traits according to the trends described for the genus (Maridet et al. 2009; Li et al. 2016) such as the posterior connection of the metalophule on the M1, the well-developed lingual anteroloph on the M2, and the weakly developed entoconid on the m3. However, it has also some basal morphologies (according to Li et al. 2016) such as the presence of anterocone spur on the M1, the presence of metalophulid II, sometimes connected to the metaconid, and lack of ectomesolophid on the m1. Therefore, we agree with Gomes Rodrigues et al. (2012a) that the morphology of *E. bagus* could be a representative of a different lineage from *E. caducus* and *E. asiaticus*.

*Eucricetodon jilantaiensis* Gomes Rodrigues et al., 2012a

Fig. 6

**Fig. 6:**
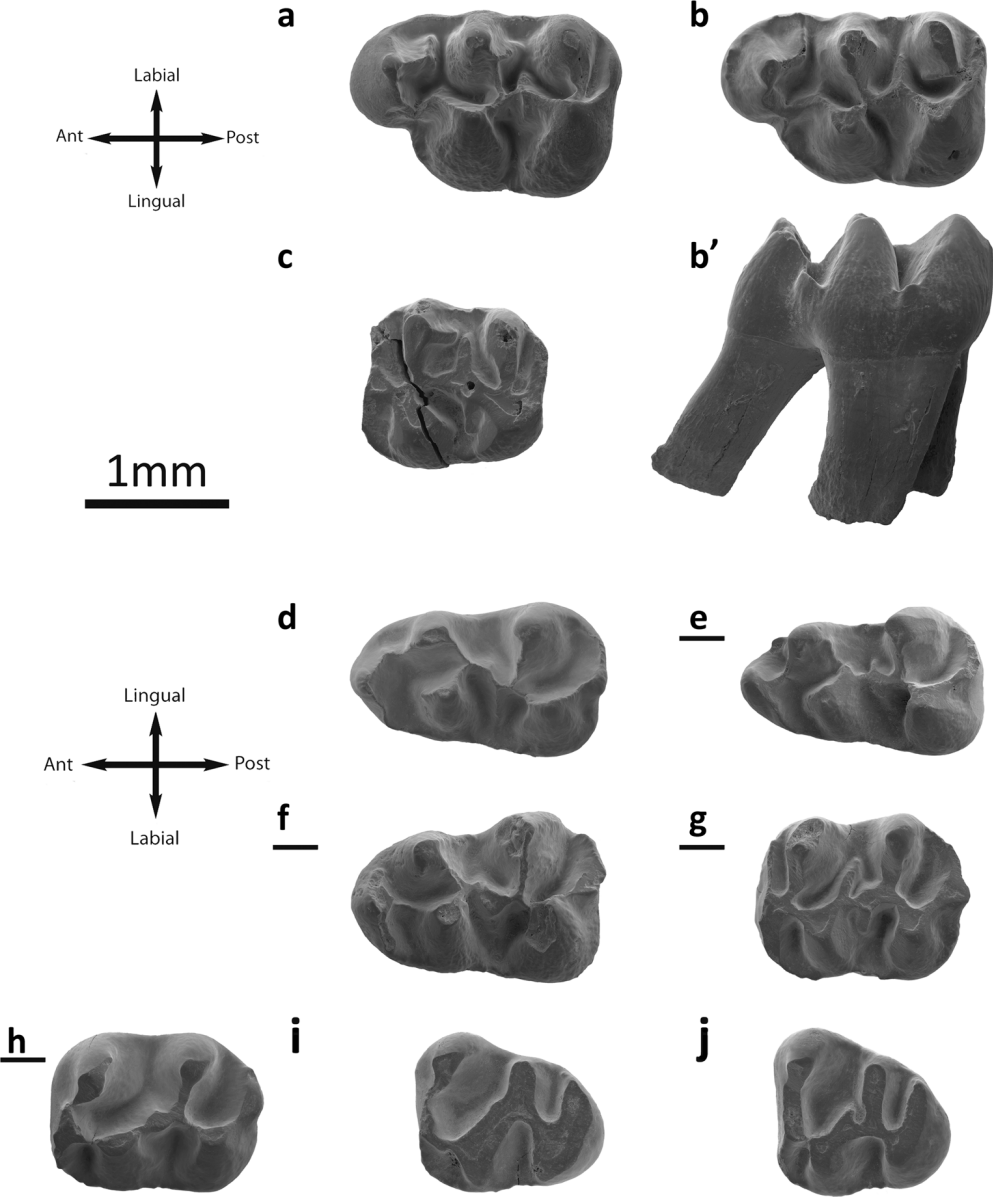
*Eucricetodon jilantaiensis* from the Valley of Lakes. **a** Toglorhoi locality, fossil layer TGW-A/2b, left M1 (NHMW2015/0336/0001). **b**, **b′** Left M1 (NHMW2015/0336/0002). **c** Left M2 (NHMW2015/0336/0005). **d** Fossil layer TGW-A/2a, left m1 (NHMW2015/0340/0011). **e** Inverted right m1 (NHMW2015/0340/0003). **f** Fossil layer TGW-A/2b, inverted right m1 (NHMW2015/0336/0009). **g** Fossil layer TGW-A/2a, inverted right m2 (NHMW2015/0340/0017). **h** Fossil layer TGW-A/2b, inverted right m2 (NHMW 2015/0336/0013). **i** Left m3 (NHMW2015/0336/0018). **j** Left m3 (NHMW 2015/0336/0016)

Synonymy

2014 cf. *Plesiodipus wangae*—Maridet et al. Table 3. p. 264. (Only for the localities: TGW-A/2a and 2b *pro parte*)

2014 *Aralocricetodon* aff. *schokensis*—Maridet et al. Table 3. p. 264. (Only for the localities: TGW-A/2a and 2b *pro parte*)

2014 *E. bagus*—Maridet et al. Table 3. p. 264. (Only for the localities: TGW-A/2a *pro parte* and TGR-C/1 *pro parte*)

**Original type locality:** UTL4 (Ulan II), early Late Oligocene, Ulantatal, Inner Mongolia, China

**Stratigraphic range:** Early Oligocene (local biozones B and C)

**Geographical range:** Central Asia

**Material:** See Table 1 (catalogue numbers NHMW2015/0336-340; NHMW2015/0267-268; NHMW2016/0010/0001; NHMW2015/0314/0001)

**Measurements:** Given in Table 2

Description

**M1** (nine specimens): The enamel is thick (Fig. 6a, b). The molar has three roots, two on the labial part and a wider one on the lingual side. The prelobe is not present. The anterocingulum is present in half of the specimens (Fig. 6b). The anterocone is large and displaced labially (Fig. 6a, b). In four molars, it is split (Fig. 6b). The labial anteroloph is always present. The anterocone spur is always present (Fig. 6a, b) and starts from the labial part of the anterocone (Fig. 6a, b); it is long and ends freely on the anterosinus in five cases; it is labially curved on the rest. Some of them reach the labial border (Fig. 6b). It is never connected to the paracone. The anterolophule is present and it connects the anterior arm of the protocone with the lingual part of the anterocone. A protostyle is present in one case (TGW-A/2a). There is no platform on the protosinus. The paracone has a weak spur; it is more developed in one molar and it is joined to the protolophule rather than the paracone (Fig. 6a). The protolophule I is absent. The protolophule II is present and connected to the entoloph. The mesoloph is always present; it is usually short, but it can be more developed (Fig. 6b) never reaching the labial border. The second mesoloph is missing. The metalophule is connected to the posteroloph in eight molars and to the hypocone in two. The posteroloph is long and reaches the metacone. The sinus is usually transversal, or slightly proverse, and narrow. The lingual cingulum is present but weak.

**M2** (13 specimens): Both lingual and labial anterolophs are well developed. The anterolophule is thick. Only one molar (TGW-A/2b) has a protolophule I that is connected to the anterolophule. The protolophule II is present on the rest (Fig. 6c) and it is connected to the entoloph. The protolophule spur is absent. The protocone lacks a posterior arm; it is connected to the entoloph only through the anterior arm. The entoloph is straight and thick; it bears a long mesoloph that never reaches the labial border. The second mesoloph is absent. The paracone spur is always present and long. The entomesoloph is present in three specimens (Fig. 6c) and it is situated on the posterior part of the sinus. The metalophule is connected to the middle part of the hypocone in six cases (Fig. 6c; TGR-AB/21; TGW-A/2a-2b); it is joined to the posterior arm of the hypocone on the rest. The mesosinus is closed by a labial cingulum. The sinus is narrow, long, and strongly proverse; it is closed by a small cingulum.

**M3** (one specimen): The labial anteroloph is present and long, and the lingual is absent. The protolophule I is present and connected to the anterolophule. The protolophule II is absent. The paracone spur is absent. The entoloph is complete; it is curved and connected to the protocone. The mesoloph is long and reaches the labial border. The posterior part of the entoloph is present and joined to the metalophule. The neoentoloph is present but incomplete. The sinus is narrow, long, and proverse. The hypocone is reduced. The posteroloph is always present and long. The mesosinus is closed by a cingulum.

**m1** (17 specimens): The anteroconid is large and situated on the longitudinal axis of the occlusal surface; it is rounded. The labial anterolophid is present but thin. The lingual anterolophid is absent in some specimens (TGW-A/2a). The anterolophulid is present only in three cases (TGW-A/2a-2b; Fig. 6f) and connected to the middle part of the anteroconid. The metalophulid I is frequently present and it is joined to the middle part of the anterocone. Only two molars lack it (TGW-A/2a; Fig. 6f). The metalophulid II is always present and connected to the posterior arm of the protoconid. The mesolophid is short or absent. The ectolophid is enlarged in its middle as a mesoconid. The ectomesolophid is usually present, frequently short, or incipient but is long in one case (Fig. 6d). The hypoconid hind arm is absent. The hypolophulid is short and connected to the ectolophid. A small stylid is present on the anterosinusid in some specimens (Fig. 6e). The mesosinusid is open or closed by a small cingulid (Fig. 6e). The sinusid is short and wide, transversally directed.

**m2** (20 specimens): Both labial and lingual anterolophids are present and well developed.

The metalophulid I is present and it is connected to the anterolophulid. The metalophulid II is absent. The posterior arm of the protoconid is well-developed and in some molars (Fig. 6g) ends freely in the mesosinusid. The mesolophid is present in some specimens (TAR-A2; TGR-C1; TGW-A/2a; Fig. 6h) and it is always short. In most teeth, the ectomesolophid is present, but it is short or incipient. The ectolophid is oblique. The sinusid is wide and transversal and open. The hypolophulid is connected where the ectolophid and the anterior arm of the hypoconid are joined. The hypoconid hind arm is absent.

**m3** (three specimens): The labial anterolophid is short but reaches the protoconid. The lingual anterolophid is also short and connected to the metaconid. In two specimens, the lingual anterolophid is absent. The metalophulid I is present and is connected to the lingual anterolophid. The metalophulid II and metalophulid spur are absent. The ectolophid is long and thin, oblique in two molars (Fig. 6i, j), and horizontal on another. The mesolophid is absent in one tooth (Fig. 6i), short and long on the other two. The ectomesolophid is always absent. The entoconid is reduced and small. The hypolophulid is attached to the anterior part of the hypoconid. The hypoconid hind arm is absent.

**Remarks:** The studied assemblages are morphological and metrically homogeneous (Table 2). The great variability found on m3 is remarkable. Two morphotypes can be distinguished: one with long mesolophid and the other with weak or absent mesolophid and shorter length. The morphology of the fossils here studied fits the diagnosis of *E. jilantaiensis* Gomes Rodrigues et al., 2012a. The most diagnostic traits are as follows: anterocone usually simple; strong anterolophule; and linked to the protocone and posterior metalophule. The M2s possess protolophule II and metalophule transversal. The m1 and m2 have metalophulid I and mesoconid. After a direct comparison with the whole collection of *E. jilantaiensis* from Ulantatal (UTL1 to 8), we have realised that the size ranges are wide enough to represent more than one species. The high intraspecific variability seems to correspond to two species. Therefore, we suggest the revision of the fossils ascribed to *E. jilantaiensis* and we compare here only with the material from the type locality (UTL 4, Ulan II early late Oligocene). Mongolian and Ulantatal material share the size, the absence of anterior protocone arm, the presence of both anterolophids, the short mesoloph, the straight sinus, and the posterior metalophule on the M1. Also, they share the presence of metalophulid II and short ectomesolophid on the m1.

*E. jilantaiensis* was only known previously in China, and it is morphologically close to *E. asiaticus*. They have been phylogenetically related (Gomes Rodrigues et al. 2012a). Both species have different proportions, illustrated by their *L*/*W* ratios (Table 2). Moreover, *E. jilantaiensis* has rounded anterocone and narrower sinuses than *E. asiaticus* and it has no second mesoloph nor platform on the protosinus or protostyle. The anterocone is split in some specimens whereas in *E. asiaticus* is always undivided and the metalophule is posteriorly directed. *E. jilantaiensis* have entomesoloph on the M2. The anterolophulid on the m1 of *E. jilantaiensis* is mostly missing; the metalophulid I is almost always present, and the mesolophid is less developed than in *E. asiaticus. E. jilantaiensis* m1 have mesoconid and no hypoconid hind arm. According to the trends described for the genus (Maridet et al. 2009; Gomes Rodrigues et al. 2012a; Li et al. 2016), all these traits confer a more derived morphological pattern to *E. jilantaiensis*; therefore, we agree with Gomes Rodrigues et al. (2012a) in their proposed phylogenetic relationship.

*Eucricetodon occasionalis* Lopatin, 1996

Fig. 7

**Fig. 7:**
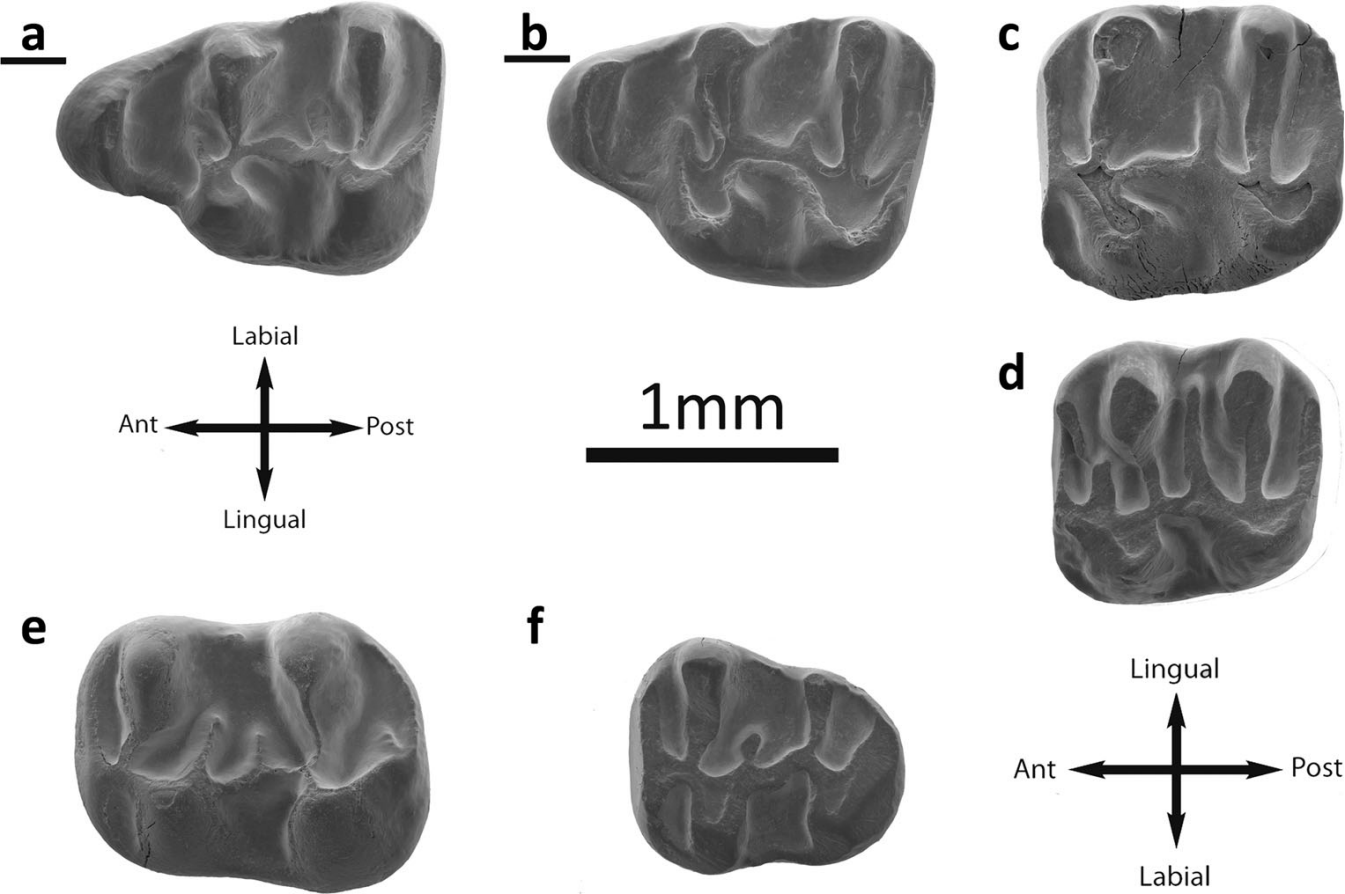
*Eucricetodon occasionalis* from the Valley of Lakes. **a** Taatsiin Gol Right locality, fossil layer TGR-AB/22, inverted right M1 (NHMW2015/0335/0001). *Eucricetodon* cf. *occasionalis*: **b** Ikh Argalatyn Nuruu locality, fossil layer IKH-A/2, inverted right M1 (NHMW2015/0334/0001). **c** Left M2 (NHMW2015/0334/0003). **d** Inverted right M2 (NHMW2015/0334/0002). **e** Left m2 (NHMW2015/0334/0005). *Eucricetodon occasionalis*: **f** Taatsiin Gol Right locality, fossil layer TGR-AB/22, left m3 (NHMW2015/0335/0002)

Synonymy

2014 *Eocricetodon* cf. *meridionalis*—Maridet et al. Table 3. p. 264. (Only for the localities: IKH-A/2 *pro parte* and TGR-AB/22 *pro parte*)

**Original type locality:** Altynshokysu, Aral Formation, Kazakhstan (Early Oligocene)

**Stratigraphic range:** Early Oligocene (local biozone B)

**Geographical range:** Central Asia

**Material:** See Table 1 (catalogue numbers *E. occasionalis* NHMW2015/0335/0001-4 and *Eucricetodon* cf. *occasionalis* NHMW2015/0334/0001-5)

**Measurements:** Given in Table 2

Description

**M1** (three specimens): The anterocone is large and crescentic; it is always simple. The labial anteroloph is well developed and it reaches the paracone (Fig. 7a, b). The lingual anteroloph is also present and connected to the protocone (Fig. 7a, b). The anterocone spur anterolophule are always absent. The anterior arm of the protocone is long and ends freely in the anterosinus (Fig. 7a, b). The protolophule I is always absent. The protolophule II is joined to the entoloph. The paracone possess a very small anterior spur. The posterior spur of the paracone is short, and in one molar, it is curved towards the labial border and reaches it (Fig. 7b). The mesoloph is always present and short; it is situated on the posterior part of the entoloph. The second mesoloph is absent. The metalophule is anteriorly directed and is connected to the anterior arm of the hypocone (Fig. 7a). When the tooth is worn, it is connected to the hypocone. The labial posteroloph is long. The anterosinus and mesosinus are closed by a thin cingulum. The sinus is slightly proverse and closed by a cingulum.

**M2** (three specimens): Both lingual and labial anterolophs are present, but the labial one is longer than the lingual one (Fig. 7c, d). The protolophule I is present in both specimens, but in one it is incomplete (Fig. 7d); it is connected to the protocone in one molar and to the anterolophule in the other (Fig. 7c). The protolophule II is present in one case (Fig. 7d) and it is attached to the posterior arm of the protocone. The entoloph is straight and long; it bears a long mesoloph that never reaches the labial border. The second mesoloph is not present. The paracone spur is always absent. The entomesoloph is absent. The metalophule is long and connected to the anterior part of the hypocone (Fig. 7c, d). The posteroloph is long and thin, and it is parallel to the metalophule and protolophule. The mesosinus is closed by a labial cingulum. The sinus is always proverse and closed by a small cingulum.

**m1:** The molar (TGR-AB/22) is too worn and no structure can be appreciated.

**m2** (one specimen): The molar is rectangular. Both labial and lingual anterolophids are present but the labial one is weaker than the lingual one. The metalophulid I is present and it is connected to the anterolophulid. The metalophulid II is absent. The protoconid hind arm is long and ends freely in the mesosinusid. The ectolophid is connected to the protoconid and it bears a mesolophid. The ectomesolophid is absent. The sinusid is wide, transversal, and short; it is closed by a small cingulid. The mesosinusid is open. The hypolophulid is anterior and connected to the posterior part of the ectolophid, clearly before the hypoconid. The hypoconid hind arm is present. The posterolophid is long and it does not display a constriction (Fig. 7e).

**m3** (one specimen): Both labial and lingual anterolophids are well developed and reach the protoconid and metaconid, respectively. The metalophulid I is present and it is connected to the anterior arm of the protoconid. The metalophulid spur is absent. The metalophulid II is always absent. The ectolophid is oblique and bears a short mesolophid that ends freely in the mesosinusid (Fig. 7f). The ectomesolophid is missing. The entoconid is well developed and it can be easily distinguished. The hypolophulid begins where the ectolophid and the anterior arm of the hypoconid are joined. The hypoconid hind arm is absent. The sinusid is transversal, wide, and closed by a cingulid. The mesosinusid is closed by a cingulid.

**Remarks:** Given the morphology and the size of the fossils, they could be assigned to the genus *Eocricetodon* Wang, 2007. However, the diagnosis of *Eocricetodon* described features that are not present on the studied material such as the mesostyles on the upper molars or the metaconid and paraconid more anteriorly located than protoconid and hypoconid on the m2. On the other hand, the material studied presents traits typical of *Eucricetodon* such as bunodont teeth, single anteroconid, and the hypoconid hind arm on the lower molars.

The small size of the studied molars distinguishes it clearly from the other species studied here. It has a size closer to *E. bagus* (Table 2; Gomes Rodrigues et al. 2012a), but it has no anterocone spur or anterolophule on the M1 as does *E. bagus*. The mesoloph is displaced posteriorly and there are no styles on the M1. The protolophule is connected to the protocone; it has no paracone spur and the metalophule is more posteriorly situated than in *E. bagus* on the M2. The m2 shows well-developed lingual anterolophid whereas is weak in *E. bagus*. The metalophulid I is connected to the anterolophulid, whereas in *E. bagus*, it is connected to the point where the anterolophulid and the anterolophid are joined. The mesolophid is less developed on the m3 than in *E. bagus*. The entoconid is well developed and it can be distinguished. The ectolophid is oblique but in *E. bagus* it is horizontal.

The Mongolian fossils present features typical of *E. occasionalis* such as low cups and narrow valleys (Lopatin 1996); an anterior lobe aligned centrally; a small unicuspid anterocone; and the short protocone spur and straight protolophule I and metalophule are found on the M1. It also displays a reduced lingual anteroloph, as well as straight longitudinal and transverse crests on the M2. The ectolophid is displaced to the labial side of the tooth on the m1. The transverse crests are curved and hypoconid hind arm is present on the m2. The protoconid and entoconid are widely spaced and the hypoconid hind arm is absent on the m3. These traits have been also found in the casts from the Altynshokysu (bone bed 2) stored at NHMW and described by Bendukidze et al. (2009). We have remeasured the casts and they have size similar to the Mongolian material. *E. occasionalis* was, until present work, only known in Kazakhstan (Lopatin 1996, 2004; Bendukidze et al. 2009). The presence of an elongated anterocone and the single protolophule on the M1 led us to describe *E. occasionalis* as a basal species. However, the scarce material does not allow to further observations.

*Eucricetodon* sp.

**Locality:** IKH-A/5 Local biozone C1 (Late Oligocene)

**m1:** NHMW2015/0264/0001. The molar has two roots. The anteroconid is rounded and well developed. The anterolophulid is not present. The metalophulid I is present and is connected to the anteroconid. The metalophulid II is also present and connected to the ectolophid. The mesosinusid is closed by a small cingulid. The sinusid is wide and short. The mesolophid is not visible, possibly due to the strong wear. The posterolophid is well developed and it reaches the entoconid. The hypoconid hind arm cannot be distinguished.

**Remarks:** Given the small size (1.42 × 0.99), this specimen could be assigned to *E. occasionalis*. However, the tooth is strongly corroded and the measurements might be slightly underestimated, the molar is strongly worn, prohibiting identification, and a taxonomical assignation cannot be done.

## Final remarks and conclusions

Both morphological and metrical features of the studied fossils led us to identify five species belonging to *Eucricetodon*. There are 11 new occurrences of *Eucricetodon* in comparison to previous studies (Maridet et al. 2014) and there is one species less than those that Maridet et al. (2014) recognised. The stratigraphical distribution of *Eucricetodon* in Mongolia remains unaltered; this genus is found in the sediments dated as early Oligocene up to the early-late Oligocene (biozones A to lower part of C1). However, the distribution of the different species has changed. *E. asiaticus* was known with certainty in biozone A and *E*. aff. *asiaticus* in biozone B (Maridet et al. 2014). This work allows us to definitively assign the specimens from biozone B to *E. asiaticus. E. caducus* was known only in biozone A and is now present also in biozone B. *E. bagus* was found in biozone C and C1, but the presence in biozone B was dubious (*E*. aff. *bagus* on Maridet et al. 2014). Our work reassigns some of those specimens from the biozone B. Finally, *E. jilantaiensis* and *E. occidentalis* are described in Mongolia for the first time.

The species of *Eucricetodon* display a combination of characters that can be used as environmental indicators. For instance, the biozone A is characterised by large-sized species with brachydont/bunodont crowns; oblique/blunt cusps, simple occlusal pattern, and low crown heights, such as *E. asiaticus* and *E. caducus*. The dental microwear analysis made in *E. asiaticus* from Ulantatal (Gomes Rodrigues et al. 2012b) indicates that its diet included a mixture of fruits and grasses with a component of animal feeding. That would imply that patches of forests were probably present (Gomes Rodrigues et al. 2012b). On the biozone B, the diversity increases with the occurrence of *E. jilantaiensis* and the smaller *E. occasionalis* and *E. bagus*. The species found here show more complicated patterns and a size decrease that, according to Bergman’s rule, could indicate an increase of temperature. The microwear analysis performed in *E. jilantaiensis* from Ulantatal reflects a diet without fruit and an increase of the potential consumption of abrasive and fibrous plants. These are more frequently found in open habitats (Gomes Rodrigues et al. 2012b). Towards the end of the biozone C and during C1, the *Eucricetodon* diversity decreases, *E. bagus* shows a trend towards size decreases for the specimens from biozone B, and the larger species disappeared. This is again consistent with Bergman’s rule and would coincide with the Late Oligocene Warming Event, recorded since 26.7 Ma (Zachos et al. 2001). More generally, the size and shape variations from the biozones A–B to C–C1 trend toward increasing crown height and more developed crests potentially indicate a general context of global cooling (Dupont-Nivet et al. 2007), local aridification, and opening of environments (Gomes Rodrigues et al. 2014).
